# Anti-metastasis activity of 5,4’-dihydroxy 6,8-dimethoxy 7-O-rhamnosyl flavone from *Indigofera aspalathoides* Vahl on breast cancer cells

**DOI:** 10.1038/s41598-024-63136-2

**Published:** 2024-05-29

**Authors:** Fatma Al-Saeedi, Peramaiyan Rajendran

**Affiliations:** 1 Department of Nuclear Medicine, College of Medicine, Kuwait University, Kuwait, P.O. Box 24923, 13110 Safat, Kuwait; 2 Department of Biological Sciences, College of Science, King Faisal University, 31982 Al-Ahsa, Saudi Arabia; 3 Department of Biochemistry, Centre of Molecular Medicine and Diagnostics (COMManD), Saveetha Dental College and Hospitals, Saveetha Institute of Medical and Technical Sciences, Saveetha University, Chennai, Tamil Nadu 600 077 India

**Keywords:** 5,4’-Dihydroxy-6,8-dimethoxy-7-O-rhamnosyl, *Indigofera aspalathoides*, Breast cancer, Wnt/β-catenin, PI3K/AKT, Cancer, Cell biology, Drug discovery

## Abstract

Breast cancer presents a significant challenge due to its high rates of illness and mortality, necessitating more effective treatment approaches. While traditional treatments offer some benefits, they often lack precision in targeting cancer cells and can inadvertently harm healthy tissues. This study aims to investigate the cytotoxic effects and molecular mechanism of 5,4’-dihydroxy-6,8-dimethoxy-7-O-rhamnosyl flavone (DDR), extracted from *Indigofera aspalathoides* Vahl, on breast cancer cells (MDA-MB-231). Through various in vitro assays including wound healing, invasion, Western blotting, and immunofluorescence, the impact of DDR on epithelial-mesenchymal transition (EMT) and metastasis was evaluated. Treatment of MDA-MB-231 cells with different DDR concentrations (0–10 µg/mL) resulted in a significant decrease in invasion and migration, accompanied by the downregulation of metastasis-related proteins including VEGF, uPAR, uPA, and MMP-9. DDR treatment also hindered EMT by upregulating E-cadherin and downregulating N-cadherin, Slug, Twist, and Vimentin. Additionally, inhibition of the PI3K/AKT signaling pathway and downregulation of the NF-кB pathway were observed. These findings highlight the potential of DDR as a valuable source of natural compounds with promising anticancer properties, offering opportunities for the development of novel cancer therapies.

## Introduction

Breast cancer is a prevalent form of cancer that affects women worldwide, constituting a significant health challenge. Annually, approximately 1.7 million new instances of breast cancer are diagnosed, representing 12% of all newly reported cancer cases^1,2^. Despite advancements in the diagnosis and treatment, post-treatment recurrence and metastasis remain significant concerns^3,4^. Emerging evidence suggests a link between epithelial-mesenchymal transition (EMT) and cancer development and metastasis^5–9^. During EMT, cancer cells undergo phenotypic changes, losing epithelial traits and gaining mesenchymal properties, thereby enhancing motility and invasiveness^5,6^. Notably, EMT involves an increase in mesenchymal markers like vimentin and a decrease in epithelial markers such as E-cadherin, leading to the disruption of cell-to-cell junctions^7^.

The significance of EMT extends beyond tumor metastasis to include the development of drug resistance, particularly through reduced E-cadherin levels in breast cancer cells resistant to taxane residues. The research underscores the role of nuclear β-catenin accumulation in facilitating EMT by disrupting the cytoplasmic β-catenin/E-cadherin complex, which is crucial for establishing epithelial junctions and hindering Wnt signaling during metastasis^8^. Additionally, EMT involves the PI3K/AKT/NF-κB mediated upregulation of Snail, promoting cell invasion and migration across various cancers. Studies have demonstrated that sustained NF-κB activation can induce EMT in breast cancer cells, with heightened PI3K activity facilitating AKT activation and NF-κB subunit p65 expression^9^. Ras activation further fuels the PI3K/AKT signaling pathway, reactivating NF-κB and inducing the expression of EMT regulatory proteins, ultimately suppressing E-cadherin expression and promoting EMT-driven metastasis^5,6^.

On the other hand, Siddha medicine, an ancient medical system originating from India, offers natural remedies with perceived benefits such as effectiveness, minimal adverse effects, affordability, and a diverse range of options. In recent decades, there has been a global trend towards accepting Siddha as a supplementary or alternative medicine, recognizing natural products as potential sources of innovative drugs^10^. For instance, *Indigofera aspalathoides* Vahl, commonly known as “sivanar vembu” in the Tamil language, has been utilized in folk medicine since approximately 300 BC for its anti-inflammatory and anti-cancer properties^11,12^. This study examines the anticancer properties and molecular mechanisms of 5,4’-Dihydroxy 6,8-dimethoxy 7-O-rhamnosyl flavone isolated from *Indigofera aspalathoides* Vahl in breast cancer cells, providing initial evidence of DDR anti-metastatic potential as a breast cancer therapy deserving further exploration and development.

## Results

### DDR inhibits breast carcinoma cells’ viability

**Figure 1:**
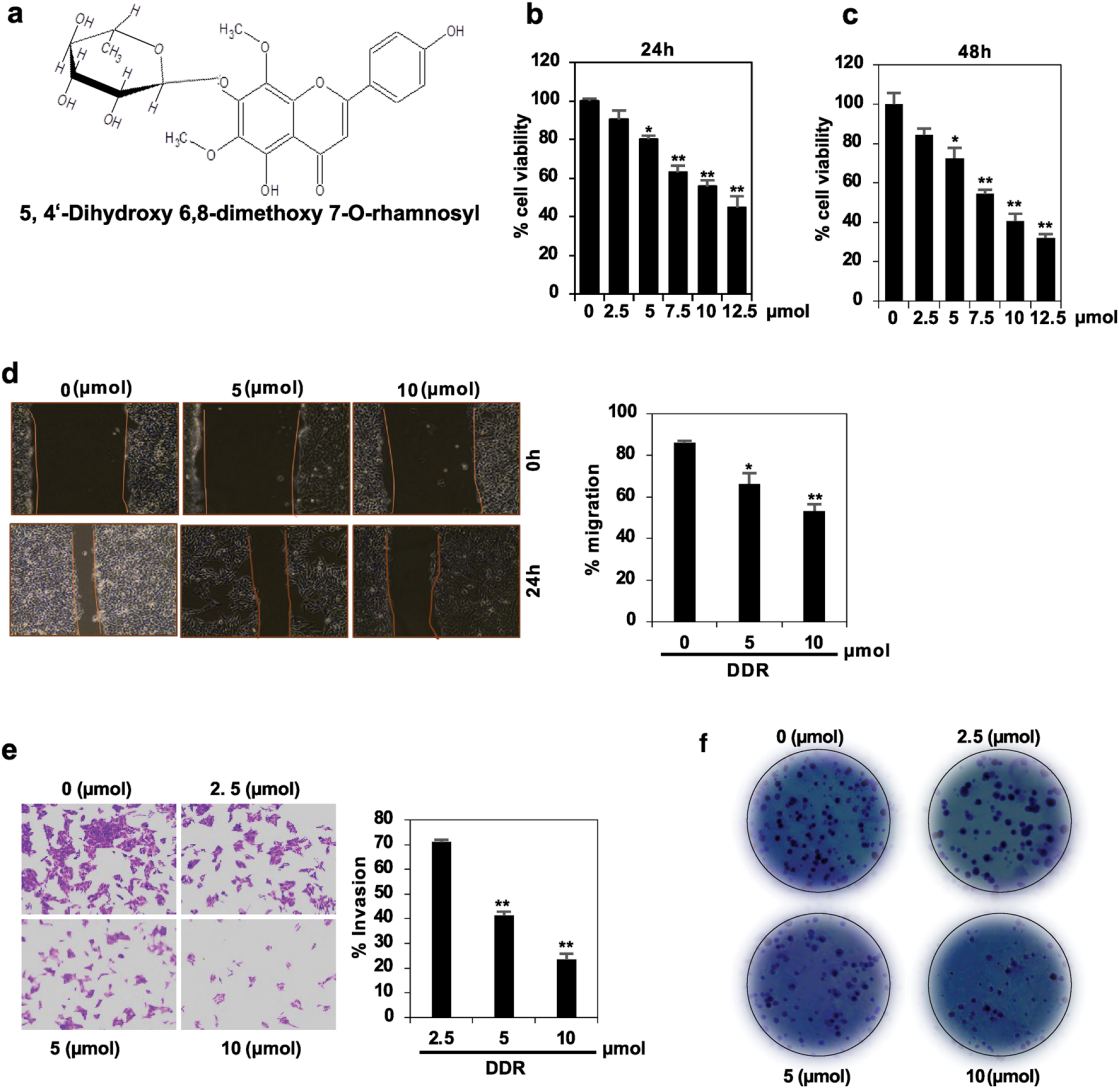
Inhibitory effect of DDR on the migration and invasion of MDA-MB-231 cells. (**a**) Chemical structure of 5,4′-Dihydroxy 6,8-dimethoxy 7-O-rhamnosyl. (**b**, **c**) MDA-MB-231 cells were treated with DDR (0–12.5 µmol) or vehicle control (0.1% DMSO) for 24 and 48 h. Cell viability was determined using the MTT assay. (**d**) Measurement of invasiveness (MDA-MB231). (**e**) Microscopic fields showing migration. (**f**) Cell Colony Formation. The results are expressed as means ± standard deviation (SD) from three independent assays. *p < 0.05 and **p < 0.01 denote statistical significance compared to control cells.

The chemical structure of DDR is illustrated in Fig. 1a. DDR impact on breast cancer cell viability was investigated using the 3-(4,5-dimethylthiazol-2-yl)-2,5-diphenyl-2H-tetrazolium bromide (MTT) assay (Fig. 1b, c). DDR demonstrated breast cancer cells (MDA-MB-231) proliferation using a dose-dependent inhibition, with IC50 values of 9.89 ± 0.41 µmol. However, minimal cytotoxicity was observed in MCF-10A (normal breast cells) (see Supplementary file). These results highlight DDR enhanced effectiveness against breast cancer cells compared to non-tumorigenic cells.

### DDR inhibits invasion and cell migration

Cancer metastasis, facilitated by EMT, involves the dissemination and migration of primary tumor cells. To assess DDR inhibition of migration through the basal membrane, in vitro wound-healing and invasion assays on DDR-treated MDA-MB-231 cells were conducted. Following 24 h of treatment, photographs of the wound area were captured at 0–10 µmol (Fig. 1d, e). DDR treatment significantly (*p* < 0.05) reduced cell migration in a dose-dependent manner.

### DDR inhibits colony formation

MDA-MB-231 cells were treated with various concentrations of DDR for 7 days to evaluate colony formation. As per the colony formation assay, MDA-MB-231 cell cloning decreased to 79.68% (Fig. 1f). DDR exhibited a dose-dependent inhibition of colony formation in breast cancer cells, indicating its effectiveness in inhibiting the formation of breast cancer colonies.

### Effect of DDR on NF-кB signaling

**Figure 2:**
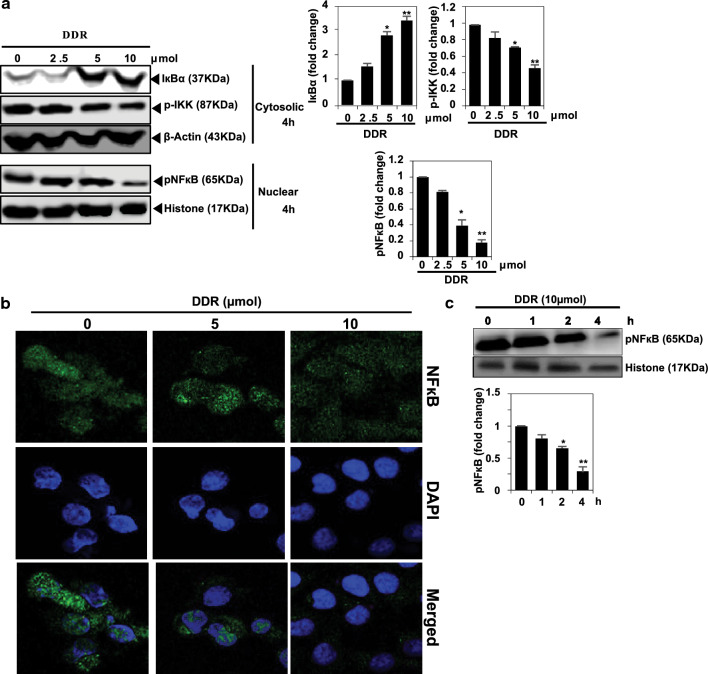
DDR inhibits metastasis in MDA-MB-231 cells by downregulating NF-κB signaling pathways. (**a**) Effect of DDR on IкBα, p-IKK, and pNF-кB activations. MDA-MB-231 cells were treated with DDR (0, 2.5, 5, and 10 µmol) for 4 h. Western blot analyses were performed on cytoplasmic and nuclear extracts. Cytoplasmic or nuclear extracts were controlled using β-actin or histone H3. (**b**) In chamber slides, cells were grown for 4 h and then fixed and permeabilized with DDR (0, 5, and 10 µmol). FITC-labeled secondary antibody was used following incubation with an anti-p65 antibody. The subcellular localization of p65 was observed using a confocal microscope at a magnification of 40×, (**c**) DDR on time dependent pNF-кB activation. The results are expressed as means ± standard deviation (SD) from three independent assays. *p < 0.05 and **p < 0.01 denote statistical significance compared to control cells.

DDR effect was investigated on NF-кB signaling and its regulatory proteins in MDA-MB-231 cells. DDR treatment inhibited IкBα protein degradation, subsequently inhibiting NF-κB activation (Fig. 2a). Immunofluorescence experiments revealed that DDR treatment dose-dependently reduced the nuclear translocation of NF-кB protein levels (Fig. 2b). These results suggest that DDR inhibits IкBα degradation, thereby suppressing nuclear activation of NF-кB. DDR time effect was investigated on NF-кB signaling and its regulatory proteins in MDA-MB-231 cells (Fig. 2c). DDR treatment inhibited successively inhibiting NF-κB activation.

### Effect of DDR on PI3K/AKT signaling in breast cancer cells

**Figure 3:**
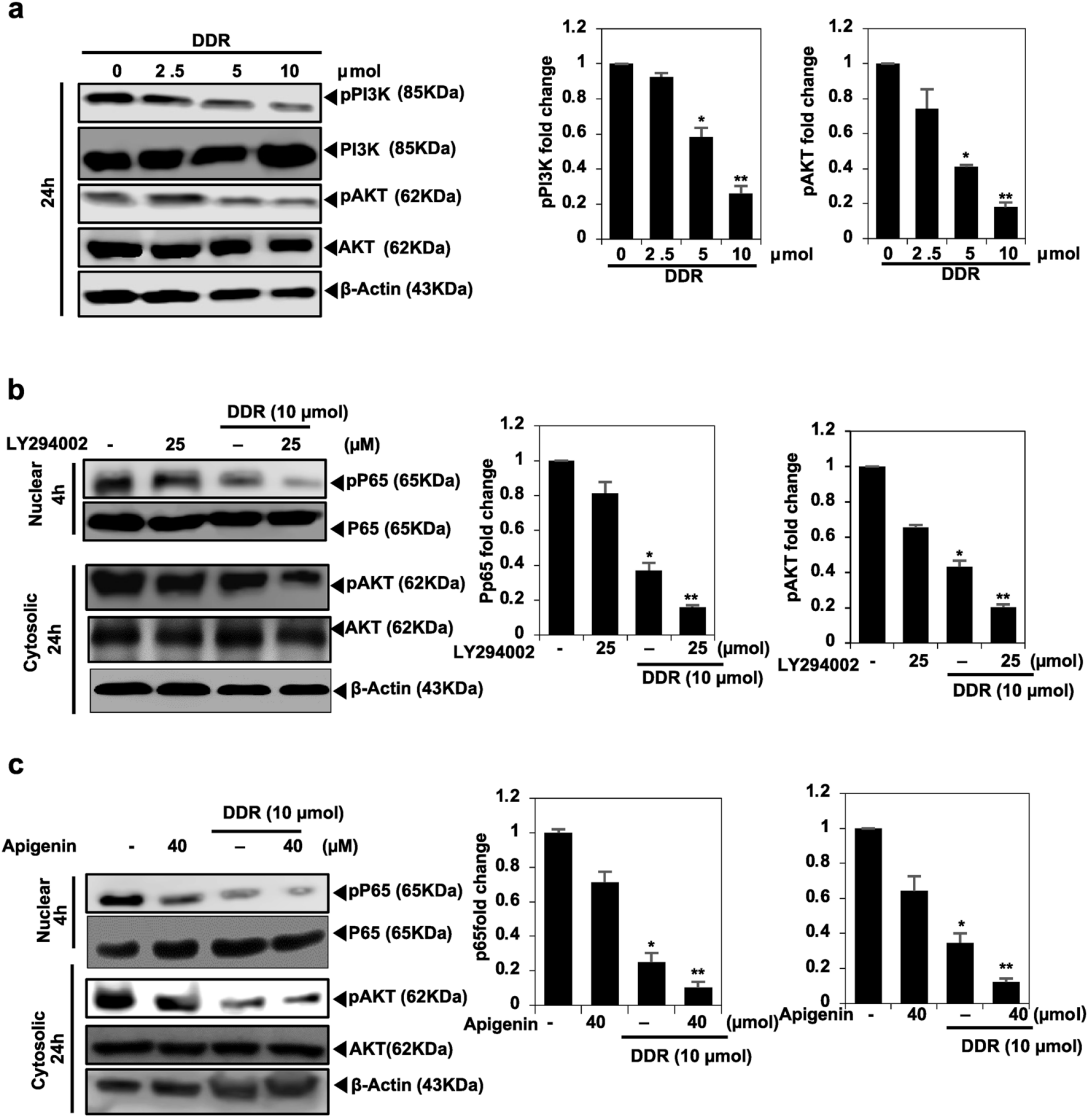
DDR inhibits PI3K/AKT signaling. (**a**) MDA-MB-231 cells were treated with DDR (0, 2.5, 5, 10 µmol) for 24 h. Immunoblotting was used to evaluate the levels of phosphorylated PI3K and AKT. Total PI3K and AKT levels served as internal controls. (**b**) The PI3K/AKT signaling pathway is downregulated by DDR, which inhibits NF-кB expression. A Western blot demonstrated that the expression of nuclear p65 for 4 h and pAKT for 24 h was inhibited following treatment with DDR (10 µmol) in the absence of PI3K/AKT inhibitor LY294002 (25 µmol). Histones and β-actin were used as internal controls. (**c**) DDR achieves downregulation of NF-κB signaling pathways. Western blot analysis revealed that treatment with DDR (10 µmol) for 4 h inhibited the nuclear p65 expression and pAKT expression for 24 h in the presence of apigenin (40 µmol), an NFкB inhibitor. Histones and β-actin served as internal controls. The results are expressed as means ± standard deviation (SD) from three independent assays. *p < 0.05 and **p < 0.01 denote statistical significance compared to control cells.

The P13K/AKT pathway is known to play a crucial role in chemoresistance and cancer cell survival^13^. DDR significantly (*p* < 0.005 and *p* < 0.01) decreased the expression of p-P13K and p-AKT in a dose-dependent manner (Fig. 3a). Treatment with LY294002, a PI3K inhibitor, further reduced the levels of p-NF-кB and p-AKT (Fig. 3b). Additionally, DDR demonstrated the inhibition of p-AKT and p-NF-кB proteins, with greater inhibition observed following apigenin pretreatment (Fig. 3c).

### EMT is suppressed by DDR by restoring pathways for E-cadherin expression in MDA-MB-231 cells

**Figure 4:**
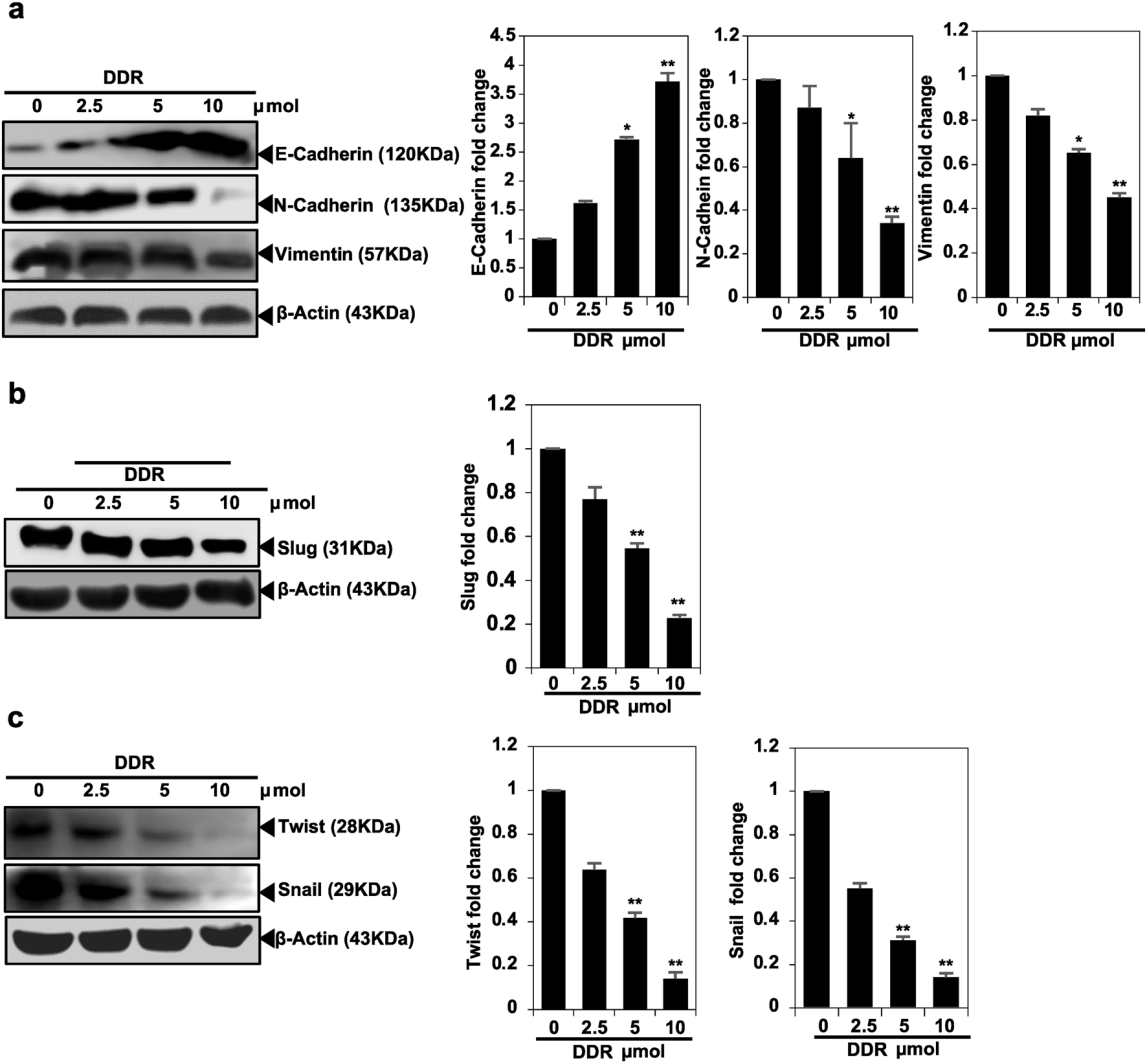
DDR inhibits EMT through the upregulation of E-cadherin signaling pathways in MDA-MB-231 cells. Cells were treated with DDR (0, 2.5, 5, and 10 µmol) for 24 h. (**a**-**c**) Western blot analysis demonstrated the expression of E-cadherin, N-cadherin, Vimentin, Slug, Twist, and Snail. β-actin served as internal control. The results are expressed as means ± standard deviation (SD) from three independent assays. *p < 0.05 and **p < 0.01 denote statistical significance compared to control cells.

Loss of the intercellular adhesion protein, E-cadherin, has been suggested to lead to invasion of surrounding tissues and metastasis, based on the observed inverse correlations between in vitro migration and E-cadherin levels^14^. It is possible to develop therapeutic approaches for metastatic breast cancer by identifying molecular strategies to inhibit E-cadherin-mediated survival. Western blotting confirmed that DDR increases E-cadherin expression. When E-cadherin expression increases because of 5,4’-Dihydroxy-6,8-dimethoxy-7-O-rhamnosyl flavone, it is due to the activation of E-cadherin transcriptional activity. Additionally, DDRtreatment reduced Slug, Vimentin, Snail, and Twist expression in a dose-dependent manner, further supporting its potential role in modulating E-cadherin-mediated pathways (Fig. 4).

### DDR downregulates matrix metalloproteinases (MMPs) and plasminogen activator proteins

**Figure 5:**
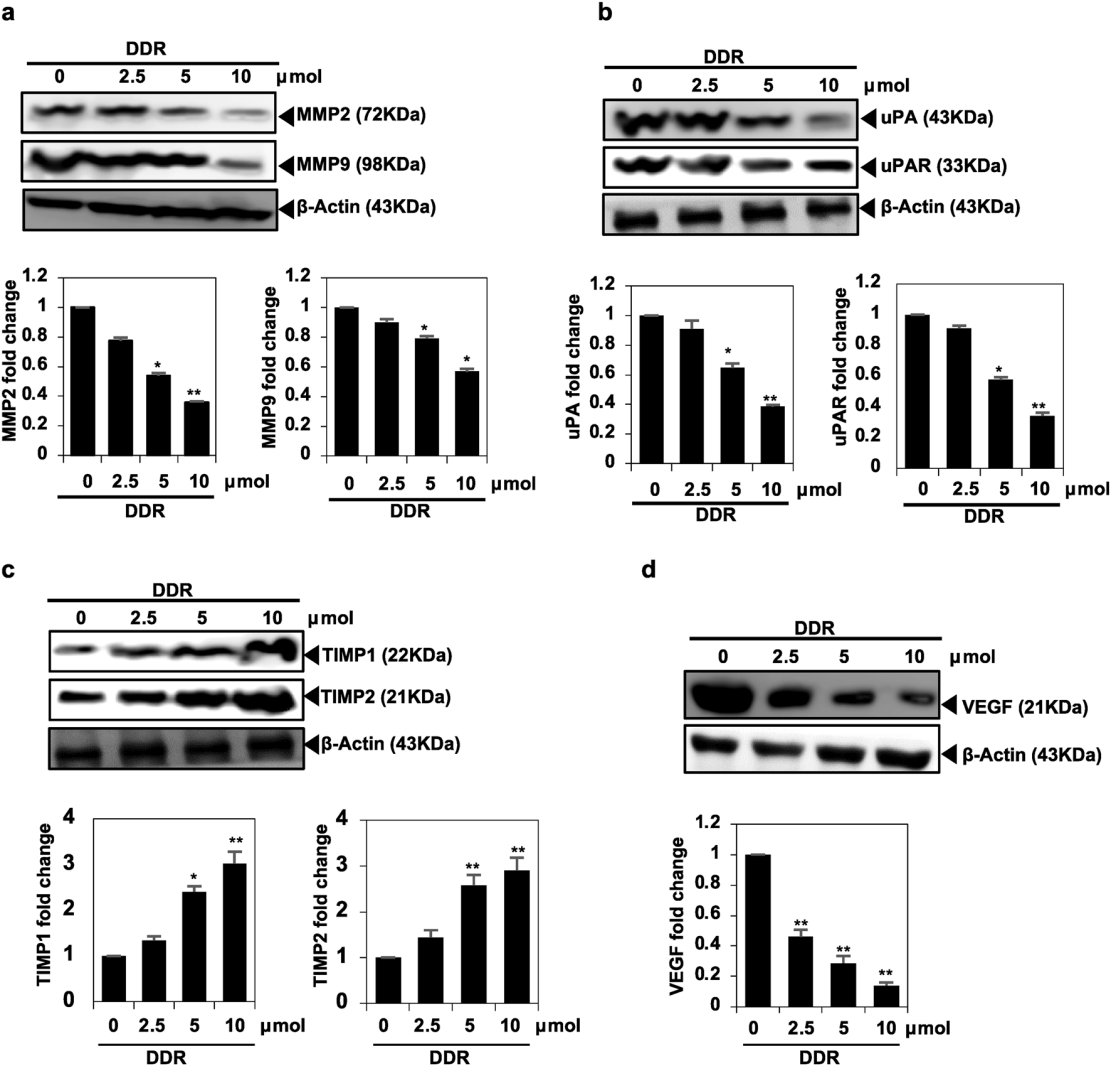
The inhibitory effect of DDR on the migration and activation of related proteins in breast cancer cells. (**a**) Activation of MMP-9 and MMP-2 analyzed by Western blot. (**b**) Activation of uPA, and uPAR analyzed by Western blot. (**c**) Activation of TIMP1 and TIMP2 by Western blot. (**d**) Angiogenesis activation protein expression VEGF. The results are expressed as means ± standard deviation (SD) from three independent assays. *p < 0.05 and **p < 0.01 denote statistical significance compared to control cells.

DDR inhibited the protein expressions of MMP-9 and MMP-2 in a concentration-dependent manner, as shown by Western blot analysis (Fig. 5a). Additionally, DDR treatment significantly downregulated the expression of uPA and uPAR in a dose-dependent manner (Fig. 5b). Further, DDR inhibited TIMPs (TIMP1 and TIMP2) expression significantly in MDA-MB-231 cells (Fig. 5c).

### DDR inhibits the angiogenesis-regulated protein expression

Targeting angiogenesis has been explored as a therapeutic strategy to inhibit tumor growth and metastasis. DDR therapy resulted in the downregulation of VEGF expression, as evidenced by Western blotting (Fig. 5d). Based on these findings, DDR may inhibit metastasis in MDA-MB-231 cells.

### DDR inhibits β-catenin signaling in MDA-MB-231 cells to attenuate EMT

**Figure 6:**
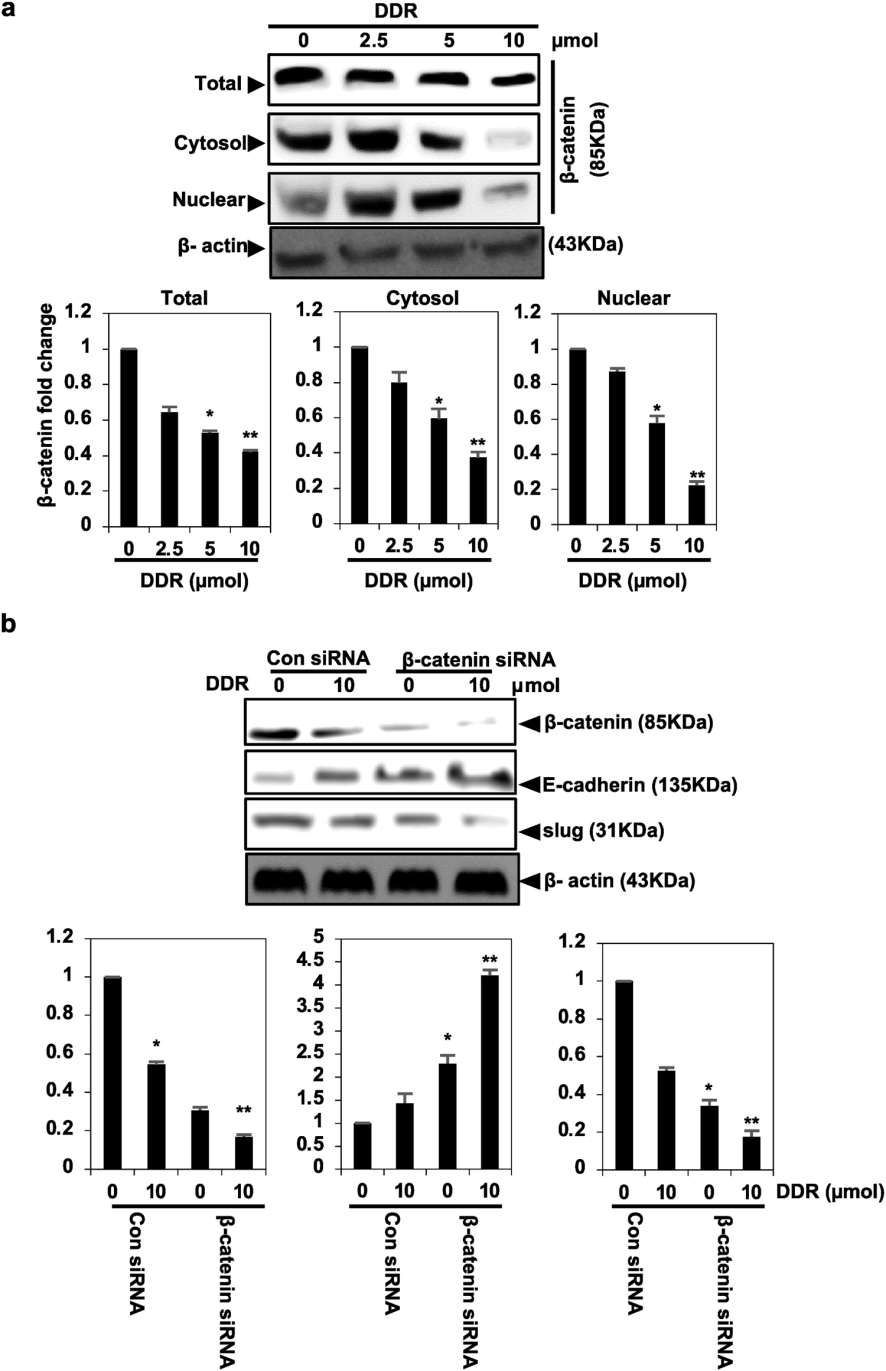
DDR inhibits EMT in breast cancer cells by downregulating the β-catenin signaling pathway. (**a**) Translocation of β-catenin from the nucleus and transcriptional activation were inhibited by DDR. Levels of β-catenin in the total nuclear and cytoplasmic fractions were determined by Western blot after 24 h of incubation with DDR (0, 2.5, 5, and 10 µmol). β-actin served as an internal loading control. (**b**) EMT was suppressed by DDR-mediated β-catenin gene silencing. Cells were transfected with RNA siRNA specific for β-catenin or a non-silencing control. After 24 h of transfection, cells were incubated with or without DDR (10 µmol). β-catenin, E-cadherin, and Slug which are transcriptional target genes of catenin, were detected by Western blot analyses. The results are expressed as means ± standard deviation (SD) from three independent assays. *p < 0.05 and **p < 0.01 denote statistical significance compared to control cells.

DDR resulted in a dose-dependent decrease in the expression of β-catenin in various cellular compartments, including the nucleus, cytoplasm, and overall cellular levels (Fig. 6a). Transfection of MDA-MB-231 cells with β-catenin siRNA confirmed alterations in E-cadherin and Slug protein expression following DDR therapy (Fig. 6b). EMT is essential for the development of cancer and depends on E-cadherin/β-catenin proteins^15^. Measuring the expression of β-catenin in cancer cells was the initial step in assessing the impact of DDR on the E-cadherin and β-catenin.

### DDR induces apoptosis in MDA-MB-231 cells

**Figure 7:**
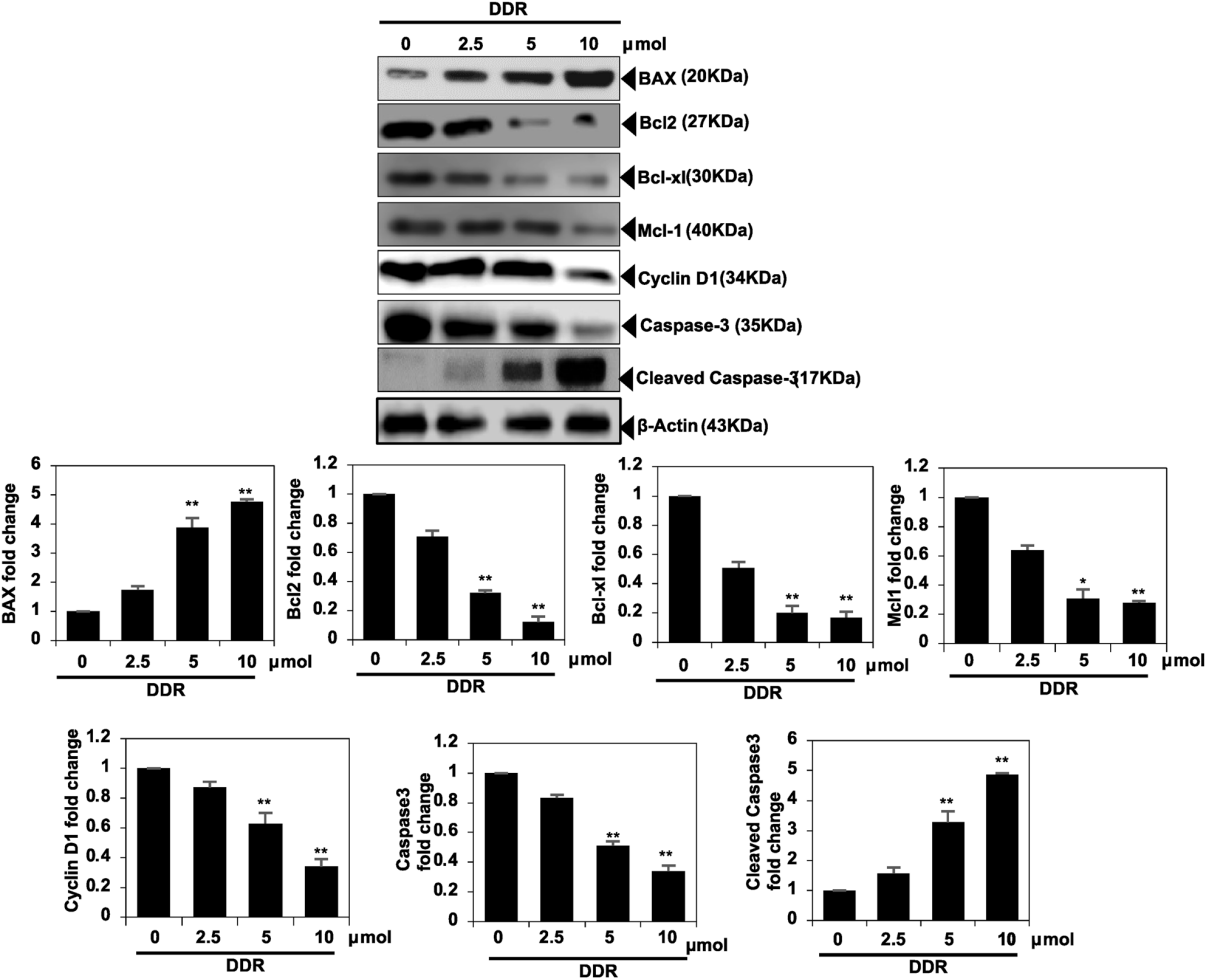
Induction of apoptosis in MDA-MB-231 cells by DDR. The cells were pretreated with DDR (0, 2.5,5, and 10 µmol) for 48 h, followed by Western blotting analysis of Bcl2, Bcl-xl, Mcl, Cyclin D1, caspse-3 and Cleaved caspase-3. The results are expressed as means ± standard deviation (SD) from three independent assays. *p < 0.05 and **p < 0.01 denote statistical significance compared to control cells.

DDR induced apoptosis in a dose-dependent manner, as evidenced by changes in Bcl2, Bcl-xl, Mcl-1, caspase-3 and cleaved caspase-3 proteins associated with apoptosis in Western blot analysis (Fig. 7). These results indicate DDR efficacy in inducing apoptosis in MDA-MB-231 cells. DDR enhances the expression of apoptotic proteins and suppresses Bcl2, Bcl-xl, and Mcl-1 proteins.

## Discussion

The progression of EMT across various cancer types crucially involves the PI3K/AKT and β-catenin signaling pathways. Additionally, researchers have identified the β-catenin pathway as a promising biomarker and potential target for cancer therapy. Studies consistently show elevated expression of mesenchymal EMT markers in breast cancer biopsies, correlating with higher recurrence rates, unfavorable clinicopathological features, lower survival rates, and greater tumor aggressiveness^16–18^. Effective therapeutic strategies are essential to address the aggressiveness of breast cancer cells and impede the spread of malignant tumors. The study aimed to assess DDR antimetastatic and anti-EMT properties and explore its underlying mechanisms in breast malignancies. MDA-MB-231 cells, known to initiate EMT in cancerous cells, exhibited downregulated E-cadherin expression and alterations in EMT-related signaling. Pretreatment with DDR restored E-cadherin protein levels and transcriptional activity, resulting in a favorable outcome. The renewal of E-cadherin was associated with DDR suppression of ß-catenin, NF-кB, and MMP-9, pivotal molecular processes in inhibiting EMT. DDR therapy effectively reduced cancer cell migration, invasion of cancer cells, colony formation, and tumor growth, indicating its potential to inhibit metastasis and EMT, possibly through modulation of specific molecular signaling pathways associated with EMT.

PI3K/AKT signaling is a commonly elevated pathway in breast cancer due to various gene mutations, particularly in estrogen receptor-positive breast cancer, as indicated by several studies^19^. Mutations in the PI3K gene, particularly PIK3CA mutations, have been linked to breast cancer and solid tumors. Breast cancer is characterized by dysregulation in multiple signaling pathways, including Wnt/β-catenin, NF-_K_B, P13K/AKT, p38, JNK, and ERK, all of which play roles in migration, EMT, metastasis, and apoptosis activation. The PI3K/AKT signaling pathways are influenced by the addition of natural compounds and combinations with classical chemotherapeutic agents^20,21^.

The inhibitor LY290042 is utilized to screen the EMT-related signaling pathways and determine their involvement in regulating EMT in DDR-treated MDA cells. The findings of our study demonstrate that DDR therapy significantly reduces the phosphorylation of PI3K/AKT. Additionally, LY290042 blocks the PI3K/AKT pathways, leading to a notable decrease in NF-κB activation and MMP-9 levels. Consistent with our findings, a study reported the inhibition of cancer cell invasion via PI3K, NF-κB, and MMP-9 signaling pathways^22^. One of its notable mechanisms involves its ability to inhibit the PI3K pathway, akin to the well-established PI3K inhibitor LY294002. By targeting this pathway, DDR exerts its antitumor effects by suppressing aberrant cell proliferation, inducing apoptosis, and inhibiting angiogenesis and metastasis. Moreover, DDR pleiotropic nature enables it to modulate multiple signaling cascades involved in cancer progression, making it a potent candidate for cancer therapy. Its efficacy as a PI3K inhibitor highlights its significance in combating cancer and underscores its potential as a valuable adjunct in cancer treatment regimens.

Various studies have demonstrated that suppressing MMP synthesis reduces the metastatic potential of cancer cells across different tumor origins, types, and stages^23–25^. MMP-9 overexpression has been linked to increased invasiveness of ovarian and breast tumors, potentially leading to decreased survival rates^26^. Conversely, flavonoids have been shown to strongly inhibit MMP-9 activity^27,28^. In our study, DDR effectively suppressed MMP-9 in MDA-MB-231 cells. Additionally, DDR treatment was found to reduce the protein levels of TIMP1, TIMP2, uPA, and uPAR in MDA-MB-231 cells, suggesting its potential anti-metastatic properties. MMP synthesis is associated with increased angiogenesis in tumor cells due to VEGF overexpression, which promotes cancer cell metastasis. Snail protein levels are elevated in breast cancer cells, resulting in the transcriptional repression of E-cadherin. Our findings indicate that DDR suppresses VEGF expression. In cancer cell development, the Wnt/β-catenin signaling pathway plays a significant role in cell fate decisions and normal cellular responses. Dysregulated or uncontrolled activation of this pathway contributes to tumor progression and metastasis in patients with breast cancer. There is growing evidence suggesting the essential role of E-cadherin in stabilizing and functioning of β-catenin^14,29^.

A decreased expression of E-cadherin leads to the separation of catenin from E-cadherin and β-catenin, followed by the translocation of β-catenin. Additionally, β-catenin binds to the TCF and LEF-1 elements, activating certain promigratory genes associated with EMT. Transcription factors, including Slug and Snail, which are transcriptional targets of β-catenin, may mediate cadherin and EMT repression. Our study demonstrated that DDR stimulated the expression of E-cadherin and caused a considerable reduction in nuclear β-catenin, as well as the protein interaction of Slug, Twist, and Snail. This was confirmed using Western blot analysis. In MDA-MB-231 cells, DDR appeared to restore E-cadherin, largely interfering with the nuclear transport of β-catenin, and thus enhancing E-cadherin expression. DDR anti-EMT effects are strongly correlated with the high expression of E-cadherin, as demonstrated by our findings. Among the most effective strategies for treating cancer, particularly MDA-MB-231, are induction apoptosis, limiting cell proliferation using chemical or biological agents, and arresting the cell cycle. Apoptosis-inducing agents are being investigated as an alternative to chemotherapy. Furthermore, we evaluated the influence of DDR on apoptosis using Western blot assays and found that DDR decreased the activation of Bcl2, Bcl-xl, and Mcl-1. Based on the Western blot assay, DDR treatment induced apoptosis in breast cancer cells by inducing caspase-3 cleavage, which is one of the major events observed during apoptosis^15^. The results of this study suggest that DDR is capable of inducing apoptosis in breast cancer cells.

**Figure 8:**
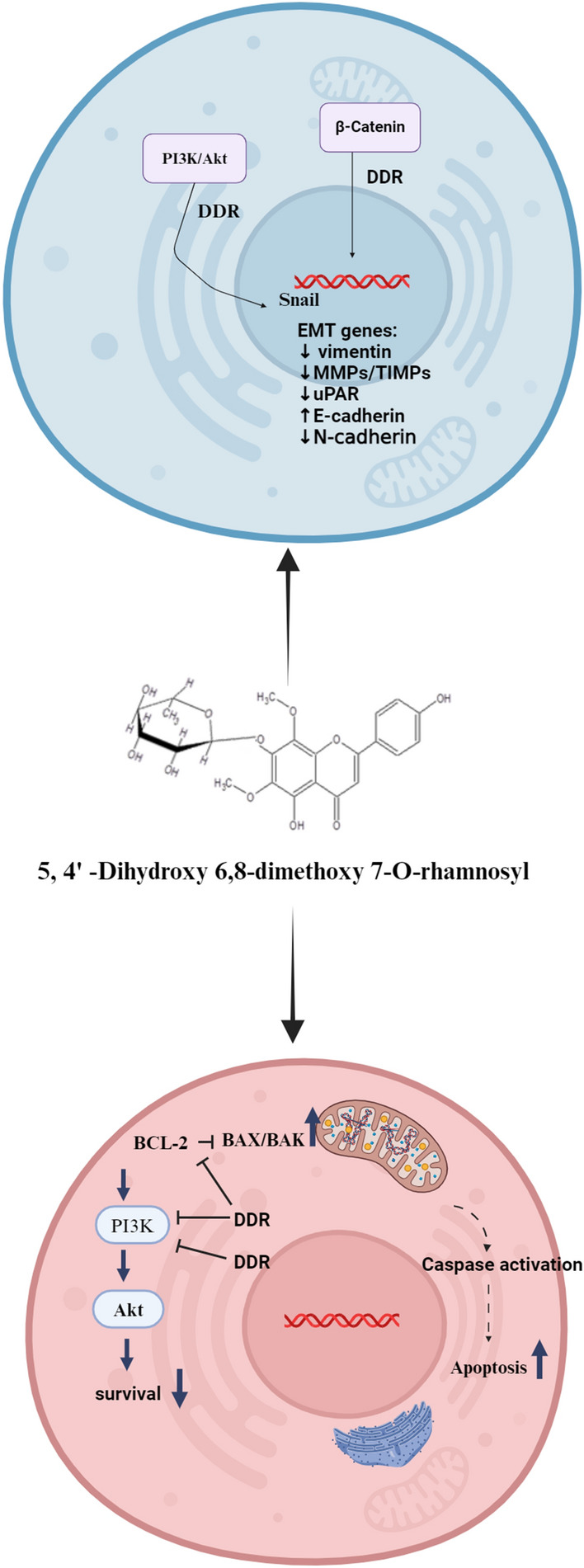
Anti-metastatic and apoptosis effect of DDR on breast cancer.

In conclusion, the study reveals that the observed effects are attributed to the modulation of PI3K/AKT, NF-κB, and β-catenin signaling pathways (Fig. 8). By elucidating the intricate molecular mechanisms involved, our findings contribute to a deeper understanding of DDR anti-epithelial mesenchymal properties and its potential therapeutic implications in breast cancer treatment. The inhibition of these specific signaling pathways suggests a multi-faceted approach of DDR in mitigating cancer progression, targeting key cellular processes such as proliferation, invasion, and metastasis. These insights not only underscore the potential of DDR as a pharmacological agent in combating breast cancer but also provide a foundation for further exploration of its clinical applications. Moving forward, future research avenues should focus on pre-clinical trials and translational studies to validate the efficacy and safety of DDR as a therapeutic intervention. Additionally, investigations into potential synergistic effects with existing treatment modalities and exploration of its application in other cancer types could expand the scope of its therapeutic utility. In summary, the outcomes presented in this research article offer a promising basis for advancing our understanding of DDR role in breast cancer therapy and open new avenues for the development of targeted and effective treatment strategies.

## Methods

### Extraction and isolation

Stems of *Indigofera aspalathoides* Vahl were collected in December from the Thanjavur district, Tamil Nadu, India, and isolation followed previously described methods^12,30^. Authentication of the stems was confirmed by Dr. G. Murthy, Scientist, Botanical Survey of India, located in Coimbatore, Tamil Nadu, India, and a voucher specimen was preserved in the laboratory (Voucher number: BSI/SRC/VMCP/13/2021). The plant extraction procedure followed the guidelines of Saveetha University Chennai and JKKN College of Pharmacy, Natarajapuram, Namakkal (DT) India. The stems (1 kg) were dehydrated, ground into a fine powder, and subjected to extraction. Alcohol extracts underwent fractionation using benzene (3 × 300 mL), diethyl ether (3 × 300 mL), and ethyl acetate (4 × 300 mL). The ethyl acetate fraction, upon concentration, yielded a yellow solid exhibiting non-homogeneity in thin-layer chromatography (TLC), thus undergoing further separation and purification via column chromatography.

#### Analysis of column chromatography

The residue from the ethyl acetate fraction (15 g) was chromatographed on a silica gel column (60–120 mesh, 300 g, 100 × 5 cm) using a gradient elution with solvents of increasing polarity. Fractions (100 mL) were collected and assessed for homogeneity by TLC. Table 1 provides details regarding the fractionation process and the characteristics of each fraction. Fractions 1–5, 6–10, 11–48, 49–64, and 65–76, when concentrated, produced residues that displayed different degrees of yellow discoloration. These residues underwent individual testing via TLC, and due to limited sample availability, no further purification was undertaken. Fractions 77–90, upon concentration, yielded a homogenous, pure yellowish compound. This compound exhibited a dark green coloration with neutral ferric chloride and a violet coloration with Molisch’s reagent. The R_f_ values of compounds in various solvent systems are given in Table 2.

**Table 1:** Chromatographic fractions of ethyl acetate concentrate of *Indigofera aspalathoides* Vahl.

Fractions collected	% Eluent composition	Remarks
1–5	100 Petroleum ether	Yellow waxy substance
6–10	90/10 Pet. Ether/benzene	Yellow waxy substance
11–48	80/20 to 10/90 Pet. Ether/benzene	Pale yellow solid
49–64	100 benzenes	Pale yellow solid
65–76	90/5 benzene /EtOAc	Yellow solid
77–90	80/20 benzene /EtOAc	Yellow solid
91–104	70/30 benzene /EtOAc	Yellow brown solid

**Table 2:** R_f_ × 100 values of DDRin paper chromatography.

Solvent system						
15% HOAc	30% HOAc	50% HOAc	60% HOAc	BAW*	Forestal^#^	PhOH^+^
Compound						
11.26	31.34	78.76	83.60	20.83	93.84	34.42

*BAW—n-butanol: acetic acid: water (4:1:5)

^#^Forestal- Acetic acid: Conc HCl: H_2_O (30:3:10)

^+^PhOH- Phenol saturated with water (3:1).

#### Acid hydrolysis of 5,4’-Dihydroxy-6,8-dimethoxy-7-O-rhamnosyl flavone

To elucidate the glycoside’s nature, compound acid hydrolysis was conducted. The glycoside solution (10 mg) in hot methanol (10 mL) was refluxed with 7% sulfuric acid for 2 h at 100 °C. The resulting aqueous solution was partitioned with ether to separate the ether-soluble aglycone and aqueous sugar.

#### Identification of the sugar

The aqueous layer underwent treatment with BaCO_3_ to eliminate excess sulfuric acid, and the filtrate, constituting the sugar portion, was then concentrated. Paper chromatography (PC) with Aniline Hydrogen Phthalate spray reagent (9.2 mL of aniline and 16 g of phthalic acid in 490 mL of n-butanol, 490 mL of ether, and 20 mL of water) was employed for sugar identification. The paper chromatography involved different solvent systems, and Table 3 provides the details of these systems along with the R_f_ values of the identified sugar.

**Table 3:** Rf × 100 values of sugar of 5,4’-dihydroxy-6,8-dimethoxy-7-O-rhamnosyl flavone.

Sugar	Developing solvents			
	BAW*	PhOH^+^	Forestal^#^	EtOAc:Pyridine: H_2_O (10:4:3)
Sugar from compound	38	55	58	54
Authentic Rhamnose	37	55	59	55

*BAW—n-butanol: acetic acid: water (4:1:5).

^+^PhOH- Phenol saturated with water (3:1).

^#^Forestal- Acetic acid: Conc HCl: H_2_O (30:3:10).

#### UV spectral characteristics of 5,4’-Dihydroxy-6,8-dimethoxy-7-O-rhamnosyl flavone

UV spectra were obtained using a spectrophotometer (Shimadzu 1601) in methanol and various shift reagents, providing λmax values. The values are tabulated in Table 4.

**Table 4:** λ_max_ Values of 5,4’-dihydroxy-6,8-dimethoxy-7-O-rhamnosyl flavone.

Solvent/shift reagents	λ_max_ (nm)
MeOH	273, 329
+ NaOMe	273, 340, 389
+ NaOAc	273, 313
+ NaOAc + H_3_BO_3_	272, 329
+ AlCl_3_	270, 348
+ AlCl_3_ + HCl	270, 300, 315, 347

#### ^1^H-NMR Spectral data of 5,4’-Dihydroxy-6,8-dimethoxy-7-O-rhamnosyl flavone

The ^1^H-NMR spectrum was recorded using an AMX 400 (400 MHz) spectrometer (Plate 1) with DMSO-d6 as the solvent, providing a complete assignment as shown in Table 5.

**Table 5:** ^1^H-NMR data of DDRin DMSO-d6.

δ H (ppm)	Signal assignment
12.95	1 H, s, 5-OH
7.89	2H, d, (J = 8.7 Hz ), H-2′ 6′
6.93	2H, d, (J = 8.7 Hz), H-3′ 5′
6.38	^1^H, s, H-3
5.15	^1^H, d, (J = 2 Hz), H-1 of rhamnose
3.9	3H, s, OCH_3_ group
3.8	3H, s, OCH_3_ group
3.0–3.75	Sugar protons
1.2	3H, d, J = 6 Hz, H-6″

#### ^13^C-NMR Spectral data of DDR

The ^13^C-NMRspectrum was recorded using an AMX 400 (100 MHz) spectrometer (Plate 2) with DMSO-d6 as the solvent, providing complete assignments as given in Table 6.

**Table 6:** Assignment of carbon in the ^13^C-NMR spectrum of the 5,4’-dihydroxy-6,8-dimethoxy-7-O-rhamnosyl flavone.

C	C-2	C-3	C-4	C-5	C-6	C-7	C-8	C-9	C-10
ppm	164.9	102.3	180.9	157.6	131.5	161.1	128.6	148.0	105.5
C	C-1’	C-2’	C-3’	C-4’	C-5’	C-6’			
ppm	120.0	127.9	115.9	160.5	115.9	127.9			
C	C-1”	C-2”	C-3”	C-4”	C-5”	C-6”	OCH_3_	OCH_3_	
ppm	98.4	70.1	69.9	71.8	69.9	18.7	60.1	56.1	

#### Characterization of active principles (5,4’-Dihydroxy-6,8-dimethoxy-7-O-rhamnosyl flavone***)***

Characterization of active principle followed by previous study^30^. The compound was obtained as a pale-yellow amorphous powder through crystallization from alcohol. UV spectra indicated intense maxima at 273 nm (band II) and 329 nm (band I), confirming its flavone nature. Chemical reactions further confirmed the presence of specific functional groups. Notably, the addition of sodium methoxide induced a significant bathochromic shift of + 60 nm in band I, indicating the presence of a free 4’-OH group in ring B. Conversely, the absence of a characteristic bathochromic shift upon the addition of NaOAc suggested that C-7 is not free, while the lack of such a shift with NaOAc/H_3_BO_3_ indicated the absence of an O-dihydroxy substituent in ring B. A consistent bathochromic shift of band II (14 nm) with AlCl_3_/HCl indicated the presence of a hydroxyl substituent at C-5 along with oxygen at the C-6 position^30^. In the ^1^H-NMR spectrum, the compound exhibited a pair of doubles in the aromatic region at δ 7.89 ppm and δ 6.93 ppm, each integrating two protons, indicating the presence of two A2B2 pattern due to protons at C-3’, C-5’, and C-2’, C-6’, respectively, of ring B of the flavone, supported by UV shift experiments and ^13^C-NMR values^31^. The violet coloration of the compound with Molisch’s reagent indicated the presence of a glycoside moiety and the position of glycosylation at C-7, as indicated by UV studies, was confirmed by the presence of an anomeric proton signal at 5.15 ppm^30^.

The rhamnosyl nature of the sugar and its attachment to the C-7 carbon were confirmed by acid hydrolysis and ^1^H-NMR studies. The ^1^H-NMR spectrum also showed a singlet at δ 6.2 ppm corresponding to the C-3 proton of the flavone skeleton, supported by the ^13^C-NMR signals at δ 164.9 (C-2), 102.3 (C-3), and a quaternary signal at 180.9 (C-4). The absence of other characteristic signals in the aromatic region of the ^1^H-NMR spectrum suggested that all the carbon atoms of ring A are substituted. The 5-hydroxy and C-6, C-7, and C-8 substitution of ring A were further supported by the ^13^C-NMR values at δ 157.6 (C-5), 161.1(C-7), 131.5(C-6), and 128.6 ppm (C-8)^30^.

The absence of signals for H-6 and H-8 in ^1^H-NMR, the downfield shift of C-6 and C-8 in the ^13^C-NMR, and the appearance of two methoxy signals at δ 60.1 and δ 56.1 ppm suggested the possibility of substitution of C-6 and C-8 by methoxyl groups. EI-MS exhibited M + m/z 330, and fragment ions at m/z 212 and m/z 118 were consistent with retro-Diels–Alder fragmentation, confirming the presence of a C-4’-hydroxyl group in ring B. Based on Rf values, UV, 1H-NMR, 13C-NMR, and EIMS spectral studies, the structure of DDR was a 5,4’-dihydroxy-6,8-dimethoxy flavone skeleton.

### Cell culture and culture media

MDA-MB-231 were obtained from the American type of Culture Collection and cultured in media containing 10% fetal bovine serum (FBS) and 1% penicillin/streptomycin at 37 ºC in 5% CO_2_.

### Cell viability assay

Cell viability is evaluated using an MTT assay. MDA-MB-231 cells (4 × 10^5^ cells/well) were plated in 12-well plates and subjected to various treatments. After treatment, the cells were incubated with MTT (0.5 mg/mL) in phosphate-buffered saline (PBS) for 2 h. Formazan crystals were dissolved in DMSO (400 μL), and absorbance at 570 nm was measured using a microplate reader (BioTek Instruments, VT, USA) to determine cell survival.

### Wound healing assay

In vitro wound healing analysis assessed cell migration. MDA-MB-231 cells (1 × 10^4^ cells/well) were cultured in 12-well plates and a pipette tip was used to scratch the cell monolayer. After scratching, cells were cultured for 24 h in an FBS-supplemented medium with the drug. Cell migration into the wounded area was observed using optical microscopy (200× magnification), and the area of the wound was quantified using Image-ProPlus (Media Cybernetics, MD, USA).

### Invasion

Cell invasion was assessed using BD Matrigel invasion chambers. 10 µL of Matrigel-coated (8 µm) polycarbonate membrane filters were seeded with cells (200 µL) treated with or without DDR in a serum-free medium. After 24 h at 37 °C, non-migrating cells were removed, and invading cells were fixed, stained with Giemsa stain, and quantified by manual counting under 200X light microscopy. Each experiment was replicated three times.

### Colony formation

Cell proliferation was evaluated using a colony formation assay. MDA-MB-231 cells were seeded in 6-well plates and treated with DDR for 7 days. Colonies were fixed with 4% paraformaldehyde for 2 h, stained with 0.1% crystal violet (750 µL/well) for 15 min, and observed under an inverted microscope after washing with PBS.

### Cytoplasmic and nuclear extraction

Cells were harvested at approximately 80% confluency and washed twice with ice-cold phosphate-buffered saline (PBS). Subsequently, cells were lysed in cytoplasmic extraction buffer containing 10 mM HEPES (pH 7.9), 10 mM KCl, 0.1 mM EDTA, 0.1 mM EGTA, 1 mM DTT, and protease inhibitor cocktail. Following a 15-min incubation on ice, lysates were centrifuged at 12,000×*g* for 10 min at 4 °C to pellet nuclei. The supernatant containing cytoplasmic proteins was carefully transferred to a fresh tube and stored at −80 °C for further analysis.

### Nuclear extraction

Pelleted nuclei obtained from the cytoplasmic extraction step were resuspended in nuclear extraction buffer containing 20 mM HEPES (pH 7.9), 400 mM NaCl, 1 mM EDTA, 1 mM EGTA, 1 mM DTT, and protease inhibitor cocktail. The suspension was vortexed briefly and incubated on ice for 30 min with intermittent vortexing. Following centrifugation at 14,000×*g* for 15 min at 4 °C, the supernatant containing nuclear proteins was collected and stored at −80 °C for subsequent experiments.

### Protein quantification

Protein concentrations in cytoplasmic and nuclear extracts were determined using the Bradford protein assay. Bovine serum albumin (BSA) was used as a standard, and absorbance was measured at 595 nm using a microplate reader.

### Immunofluorescence staining

MDA-MB-231 cells were cultured in 8-well plates at a density of 1 × 10^4^ cells per well and treated with the drug for 24 h. The cells underwent a 15-min processing step with 2% paraformaldehyde, followed by permeabilization using Triton X-100 (0.1%) for 10 min. After washing and blocking with a solution containing 10% FBS/PBS, the specimens were treated with anti-p65 primary antibodies in a solution containing 1.5% FBS. Next, the cells were incubated with secondary antibodies conjugated with fluorescein isothiocyanate (FITC), diluted in a solution containing 6% bovine serum albumin (BSA) and PBS, for 1 h. Subsequently, the cells were stained with a solution of 1 μg/mL 4′,6-diamidine2′-phenylindole dihydrochloride (DAPI) for 5 min followed by rinsing with PBS and distilled water. Ethanol treatment for 5 min ensured fixation, and the cell arrangements were finally mounted using fluoromount G. Immunofluorescence microscopy images were acquired using a Leica D6000 fluorescence microscope (Leica, Germany) equipped with an AxioCam HR (Carl Zeiss).

### Western blot

Proteins (30 µg) were separated by sodium dodecyl sulfate–polyacrylamide gel electrophoresis (SDS-PAGE gel) and transferred to a membrane. The gel was placed in a transfer buffer for 15 min, and a transfer sandwich was assembled to prevent air trapping. The sandwich components, including the gel and membrane, were loaded into the transfer unit and allowed to transfer from the gel to the membrane at 30 V for 3 h. After a quick rinse with PBS, the membrane was incubated at room temperature in a blocking solution for 1 h. Following blocking, the membrane was rinsed with PBS and exposed to a primary antibody of interest for 2 h at room temperature. Subsequently, the membrane was rinsed again with PBS, and the secondary antibody was applied for 1 h. After applying antibodies, the membrane was rinsed with PBS and soaked in a developing solution to visualize protein bands.

### Statistics

Experiments were conducted in triplicate, and the results were expressed as mean ± standard error. Statistical analysis was done with SPSS 10.0 (SPSS Inc., USA). Dunnett’s test was used to determine the significance of data when comparing pairs of data.

### Supplementary Information

Supplementary Information.

## Data Availability

All data relevant to the study is included in the article.
