# Supplementary material for: Anti-metastasis activity of 5,4’-dihydroxy 6,8-dimethoxy 7-O-rhamnosyl flavone from Indigofera aspalathoides Vahl on breast cancer cells
Source: Sci Rep. 2024 May 29;14:12349. doi: 10.1038/s41598-024-63136-2 (PMC11137080; doi:10.1038/s41598-024-63136-2)
Supplement: Supplementary file 1 — Supplementary Information. [file 41598_2024_63136_MOESM1_ESM.pdf]

Anti -metastasis activity of 5, 4′-Dihydroxy 6, 8-dimethoxy 7-O-rhamnosyl flavone from *Indigofera aspalathoides Vahl* on breast cancer cells

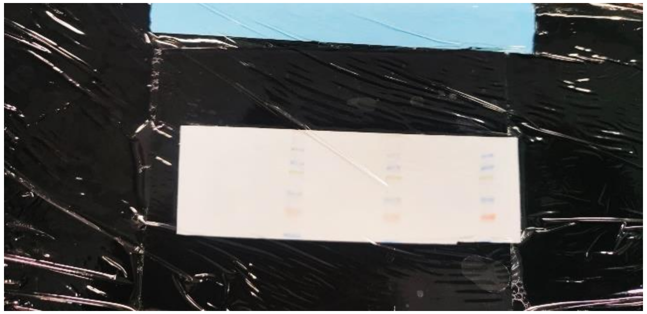

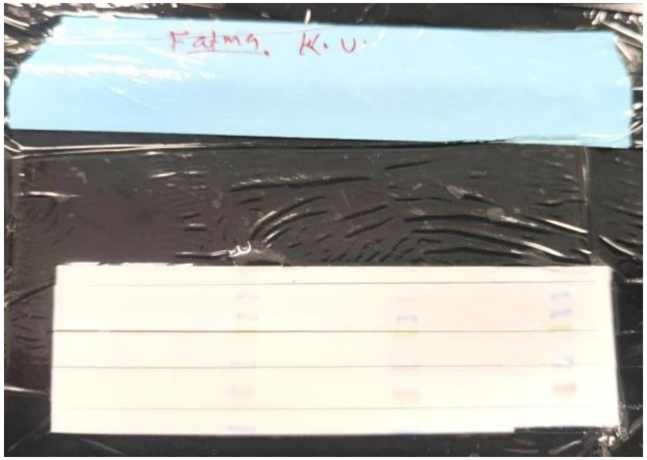

After transferring the gel onto the PVDF membrane, the membrane can be cut according to the molecular weight of specific proteins, as shown in the following image. Incubating the primary and secondary antibodies on the entire membrane would require a large amount of both antibodies and substrate. Therefore, in order to minimize the usage of antibodies and substrate, we opt to cut the membrane.

Figure 2A

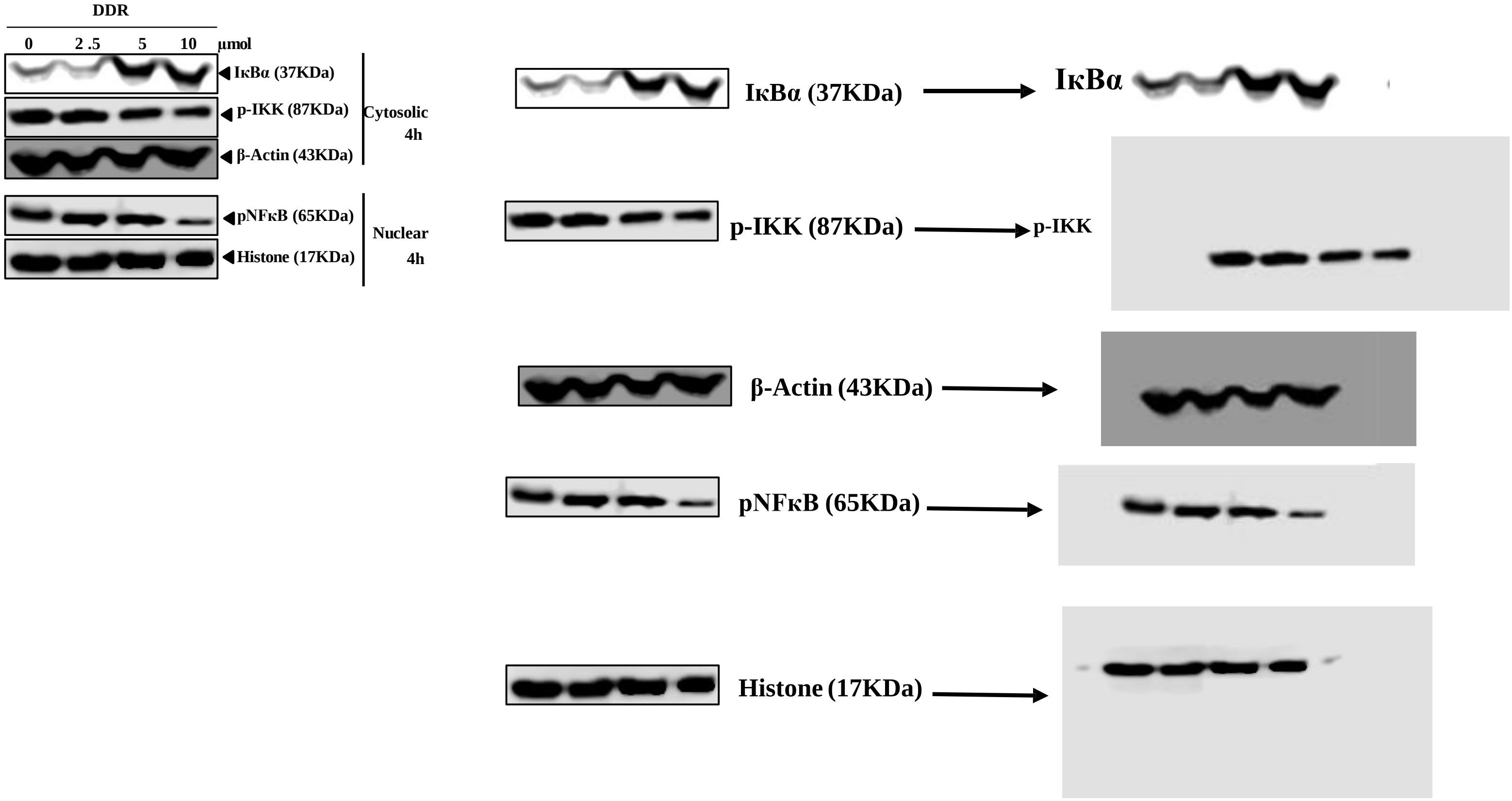

Figure 2C

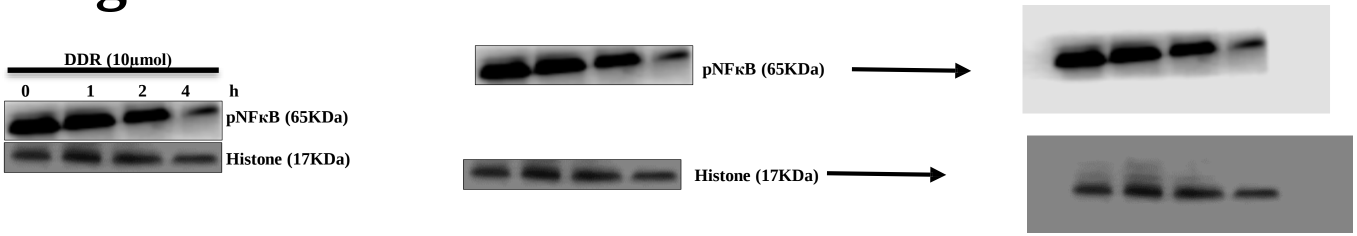

Figure 3A

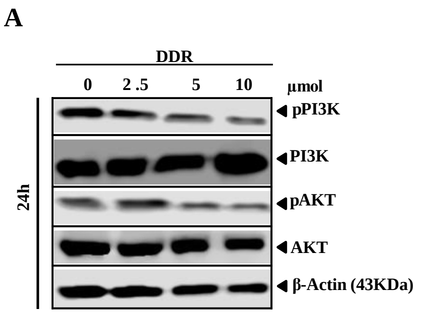
A
DDR
0
2 .5
5
10
µmol
24h
pPI3K
PI3K
pAKT
AKT
β-Actin (43KDa)

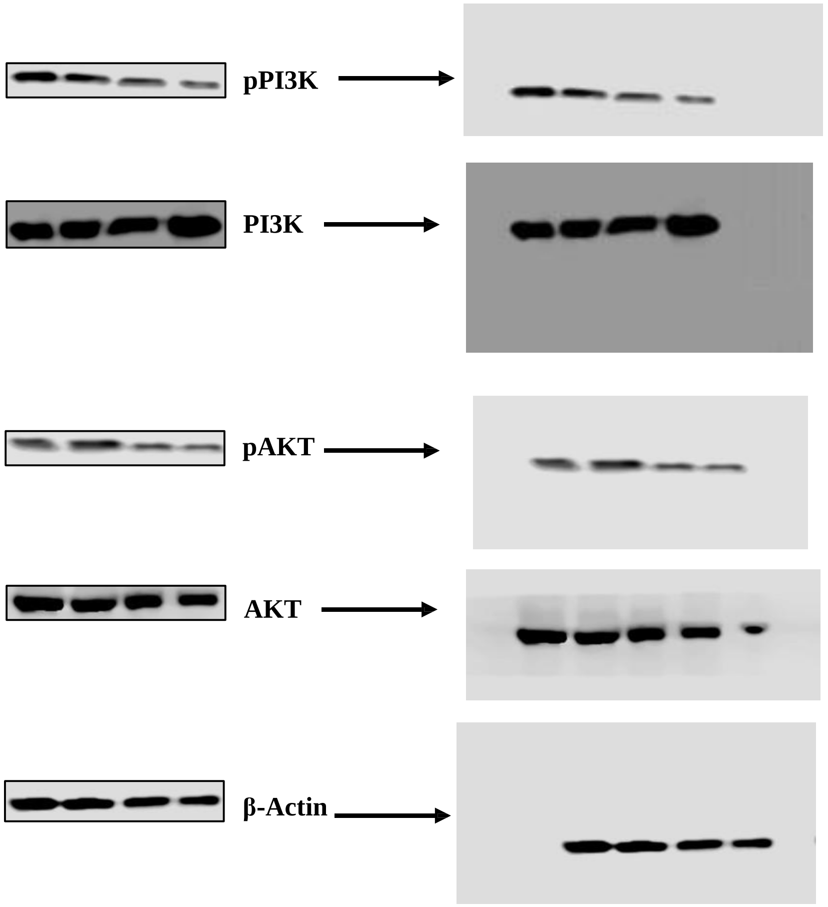
pPI3K
PI3K
pAKT
AKT
β-Actin

Figure 3B

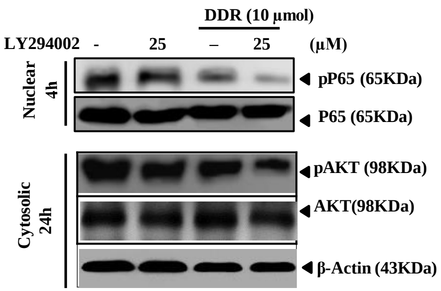

DDR (10 µmol)
LY294002 - 25 – 25 (µM)
Nuclear 4h
pP65 (65KDa)
P65 (65KDa)
Cytosolic 24h
pAKT (98KDa)
AKT(98KDa)
β-Actin (43KDa)

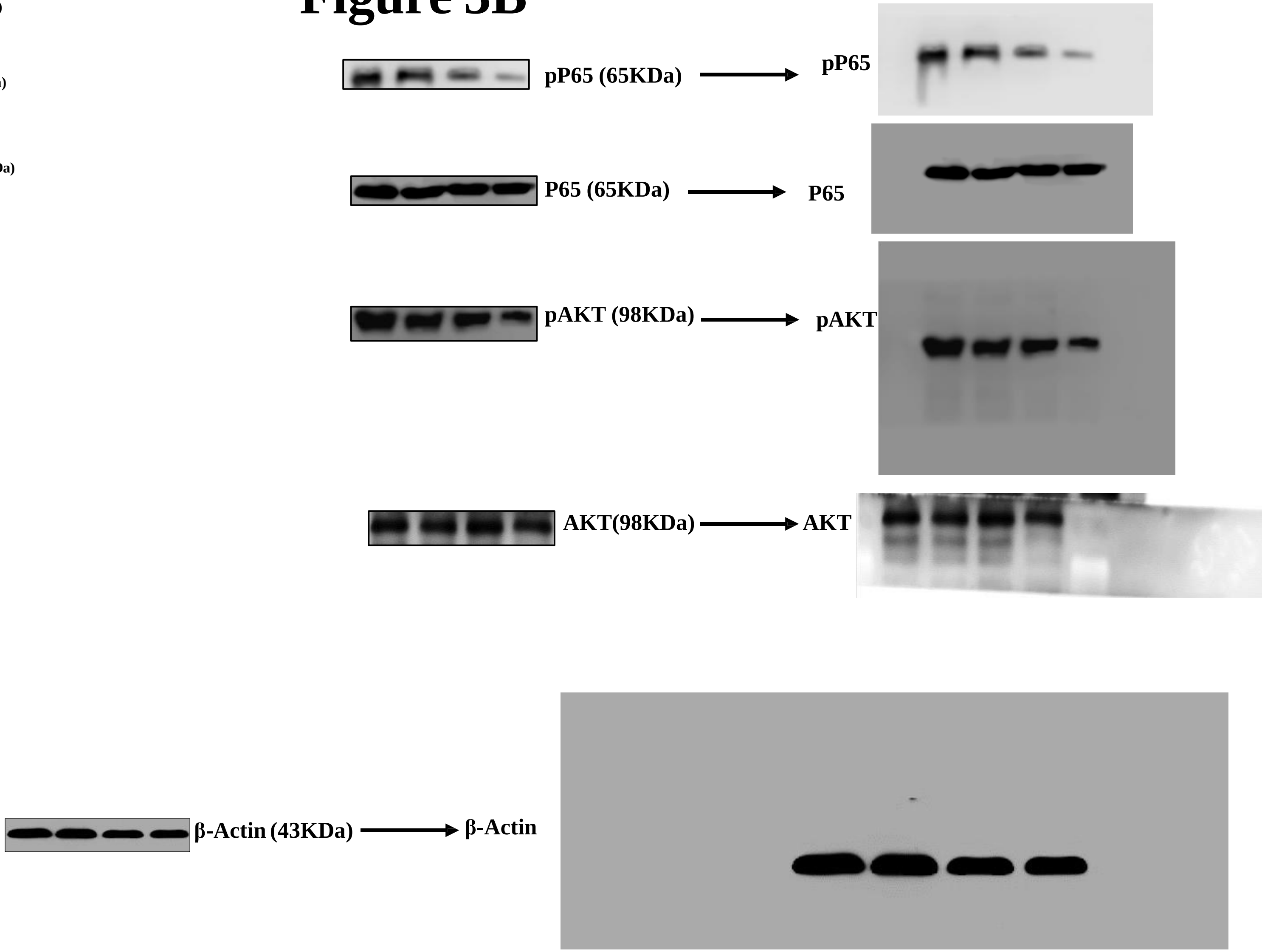

pP65 (65KDa)
pP65
P65 (65KDa)
P65
pAKT (98KDa)
pAKT
AKT(98KDa)
AKT
β-Actin (43KDa)
β-Actin

Figure 3C

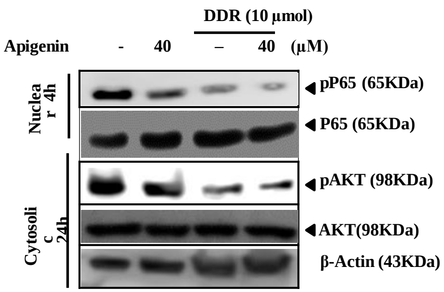
DDR (10 µmol)
Apigenin - 40 – 40 (µM)
Nuclear 4h
Cytosolic 24h
pP65 (65KDa)
P65 (65KDa)
pAKT (98KDa)
AKT(98KDa)
β-Actin (43KDa)

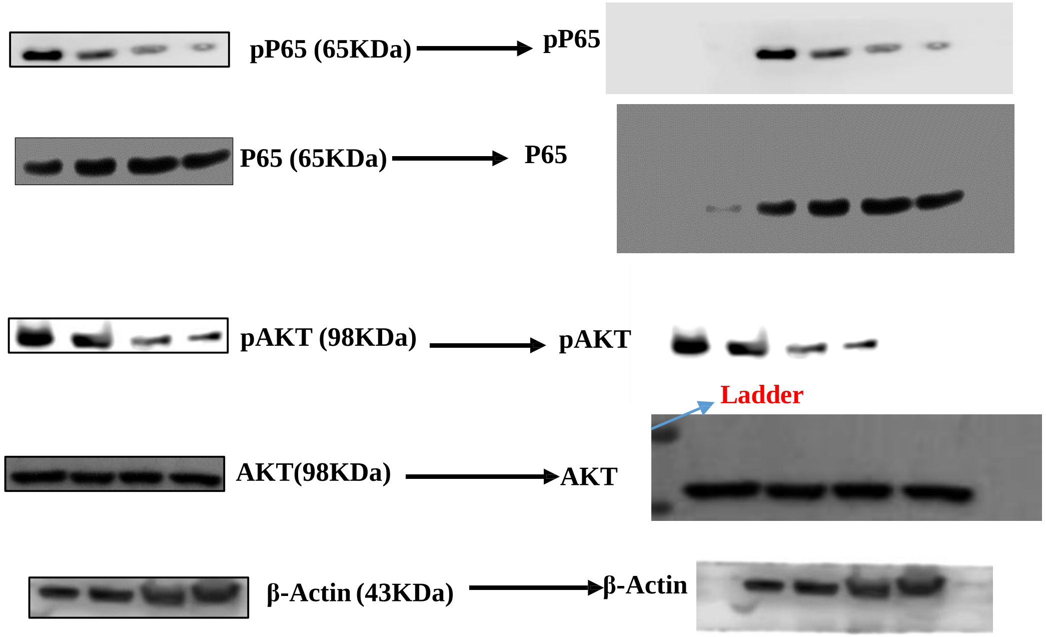
pP65 (65KDa)
pP65
P65 (65KDa)
P65
pAKT (98KDa)
pAKT
Ladder
AKT(98KDa)
AKT
β-Actin (43KDa)
β-Actin

Figure 4 A

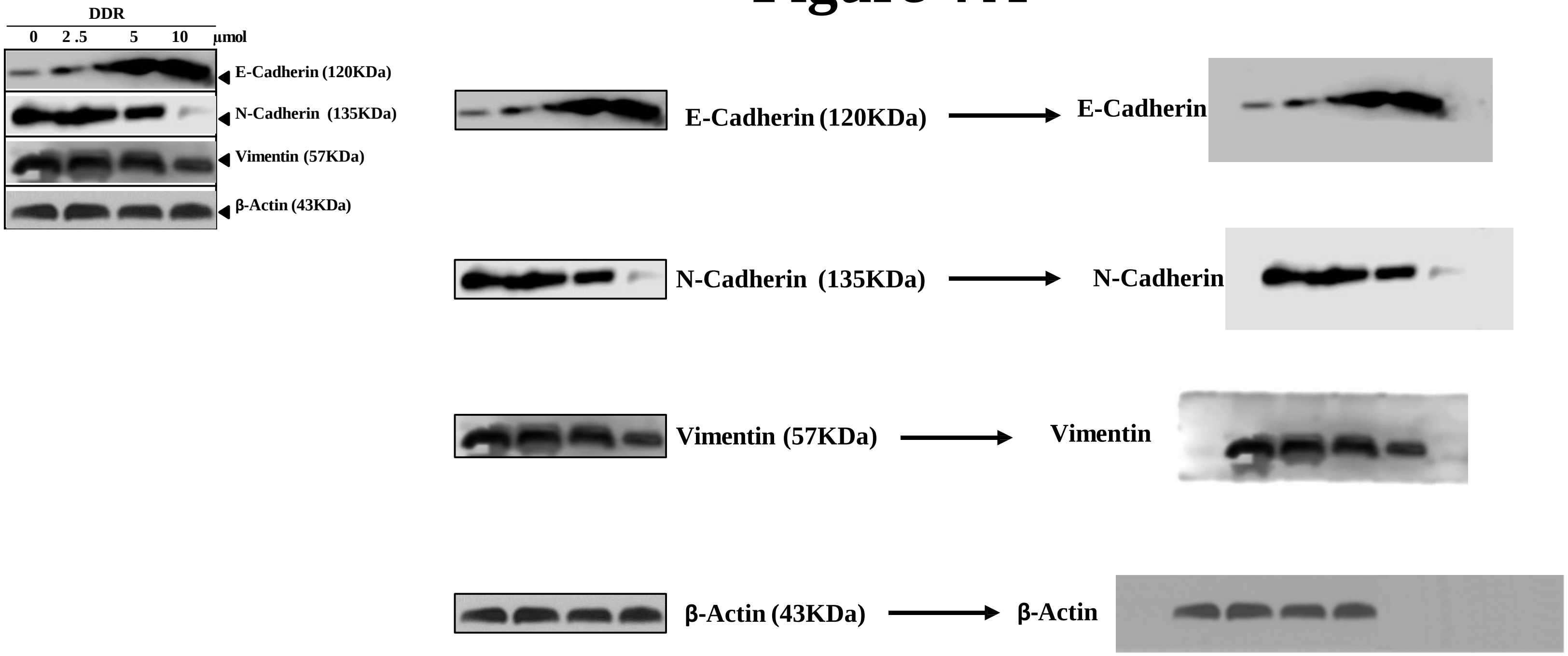
DDR
0 2 .5 5 10 μmol
E-Cadherin (120KDa)
N-Cadherin (135KDa)
Vimentin (57KDa)
β-Actin (43KDa)
E-Cadherin (120KDa)
E-Cadherin
N-Cadherin (135KDa)
N-Cadherin
Vimentin (57KDa)
Vimentin
β-Actin (43KDa)
β-Actin

Figure 4 B

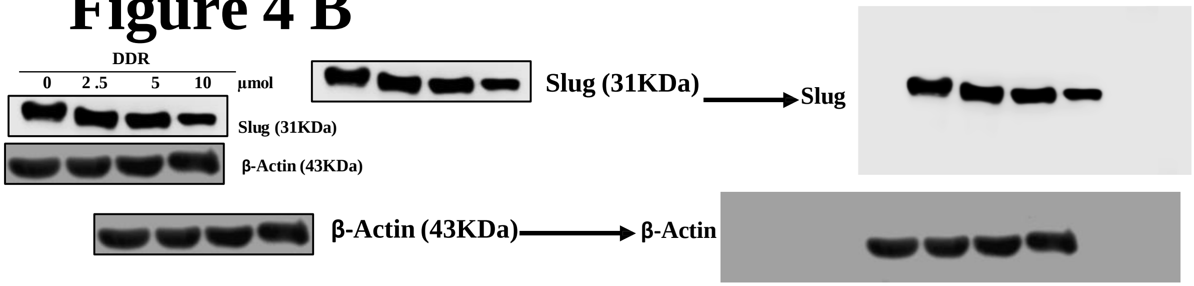

Figure 4 C

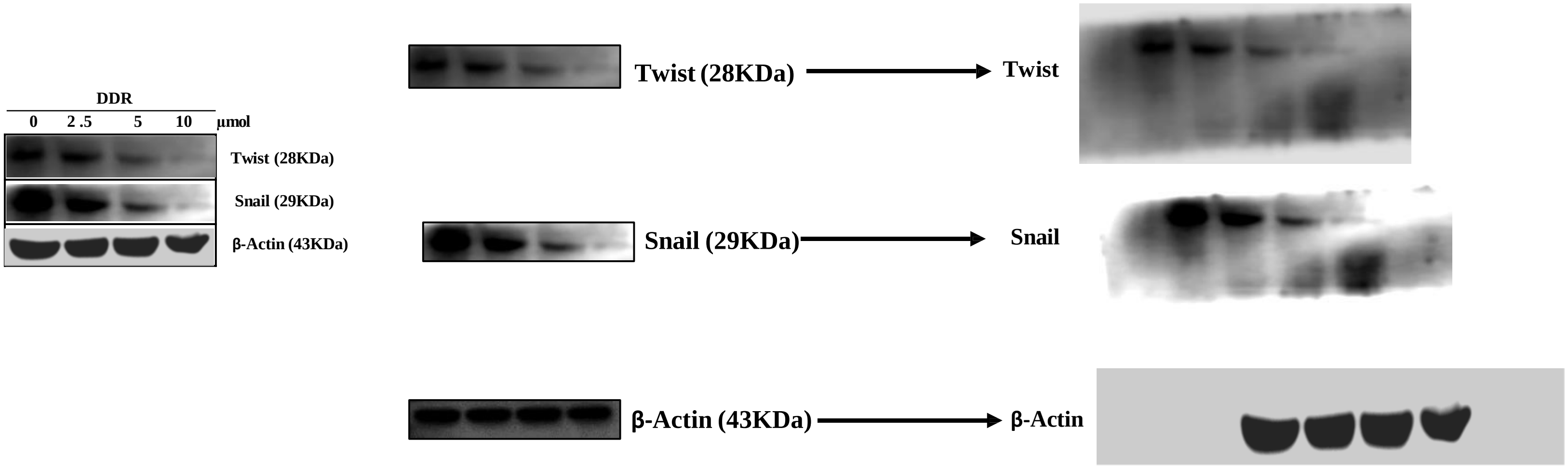

Figure 5 A

A

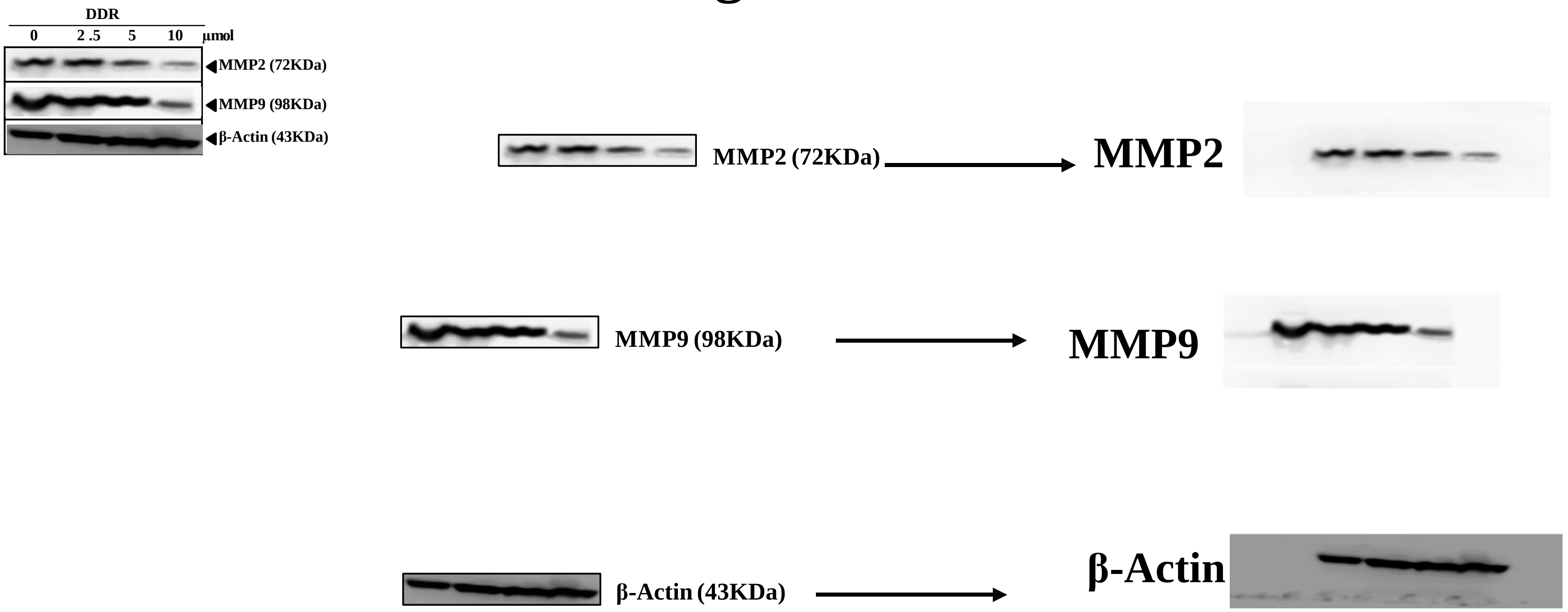
DDR
0
2 .5
5
10
µmol
MMP2 (72KDa)
MMP9 (98KDa)
β-Actin (43KDa)
MMP2 (72KDa)
MMP2
MMP9 (98KDa)
MMP9
β-Actin (43KDa)
β-Actin

Figure 5 B

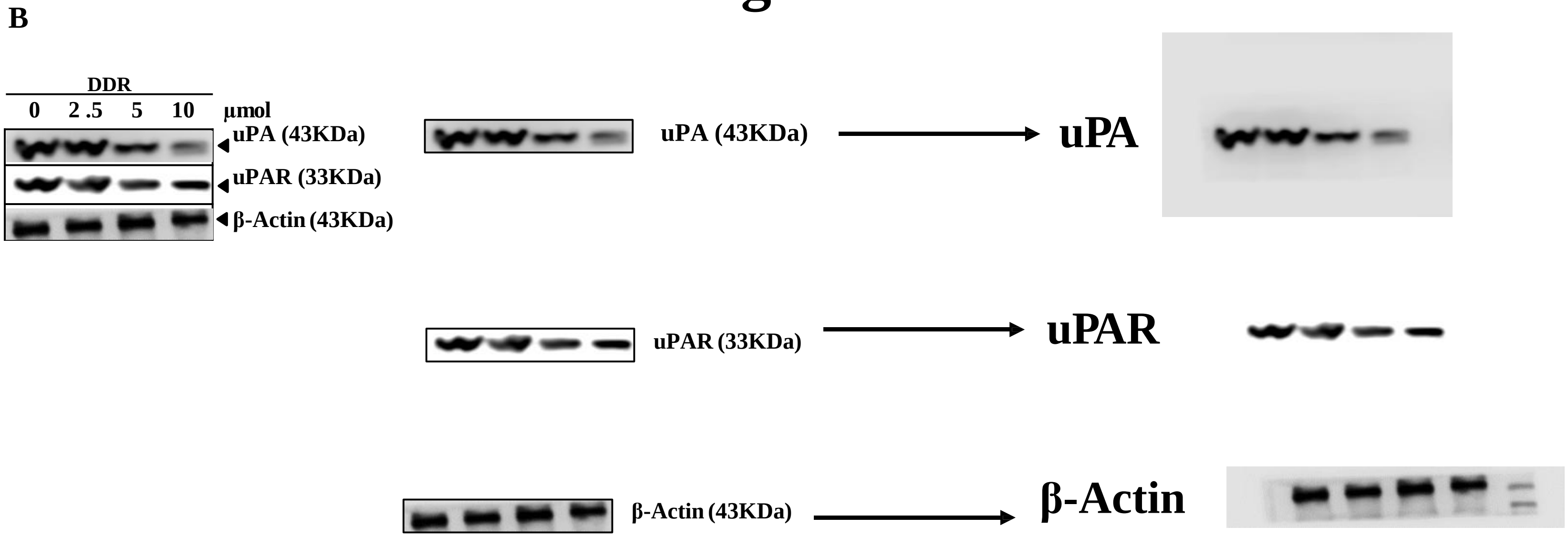
B
DDR
0 2.5 5 10 µmol
uPA (43KDa)
uPAR (33KDa)
β-Actin (43KDa)
uPA (43KDa)
uPA
uPAR (33KDa)
uPAR
β-Actin (43KDa)
β-Actin

Figure 5 C

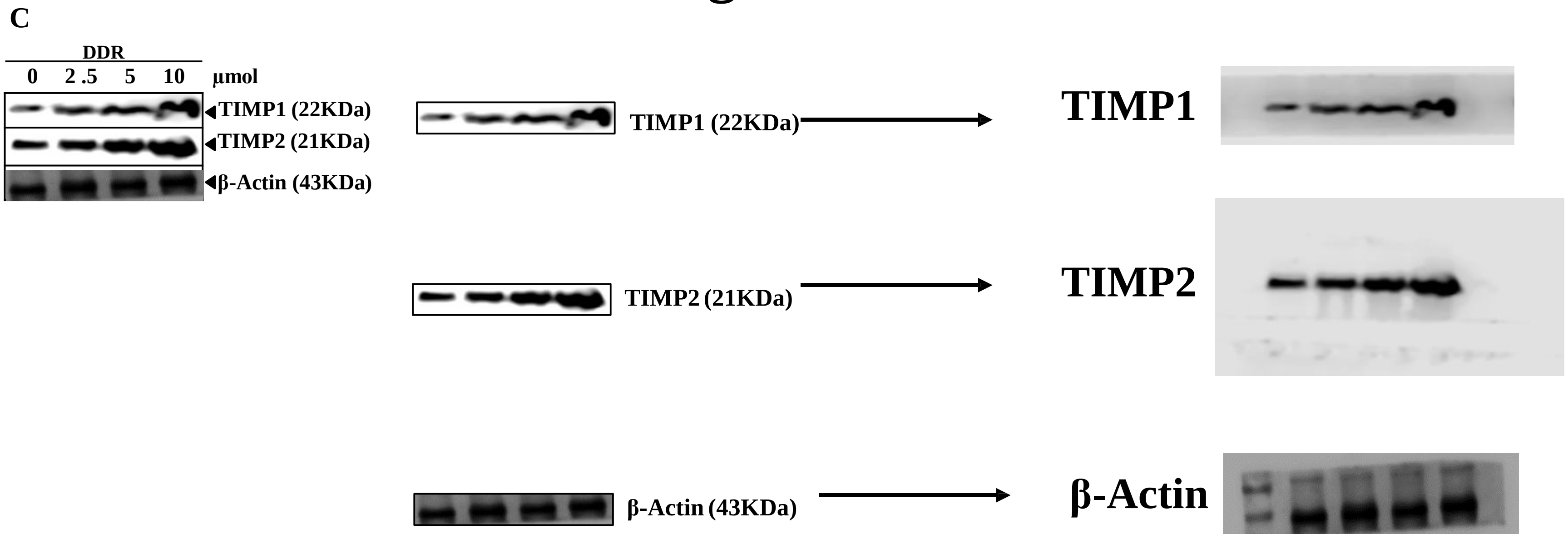

Figure 5 D

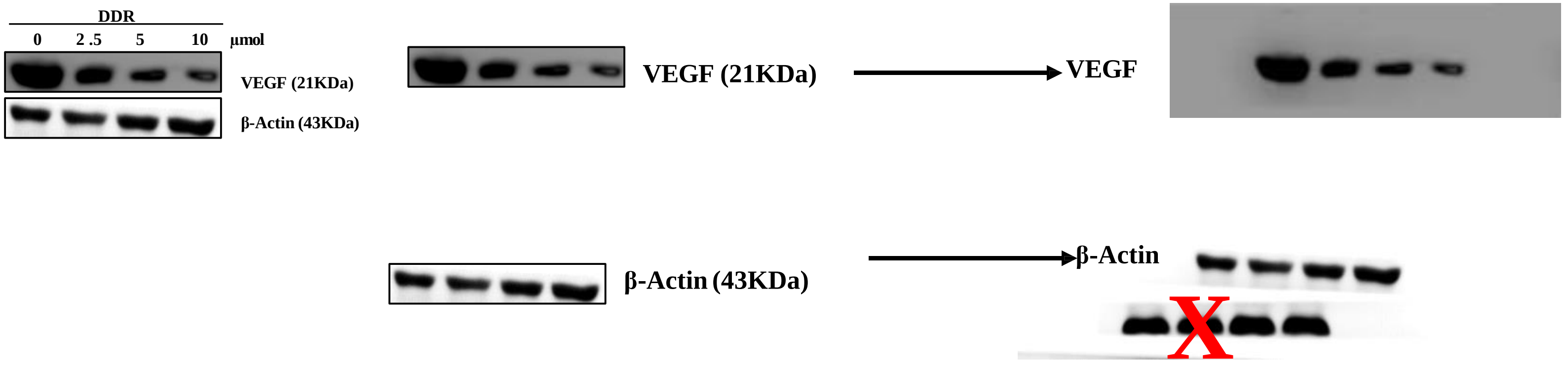

Figure 6 A

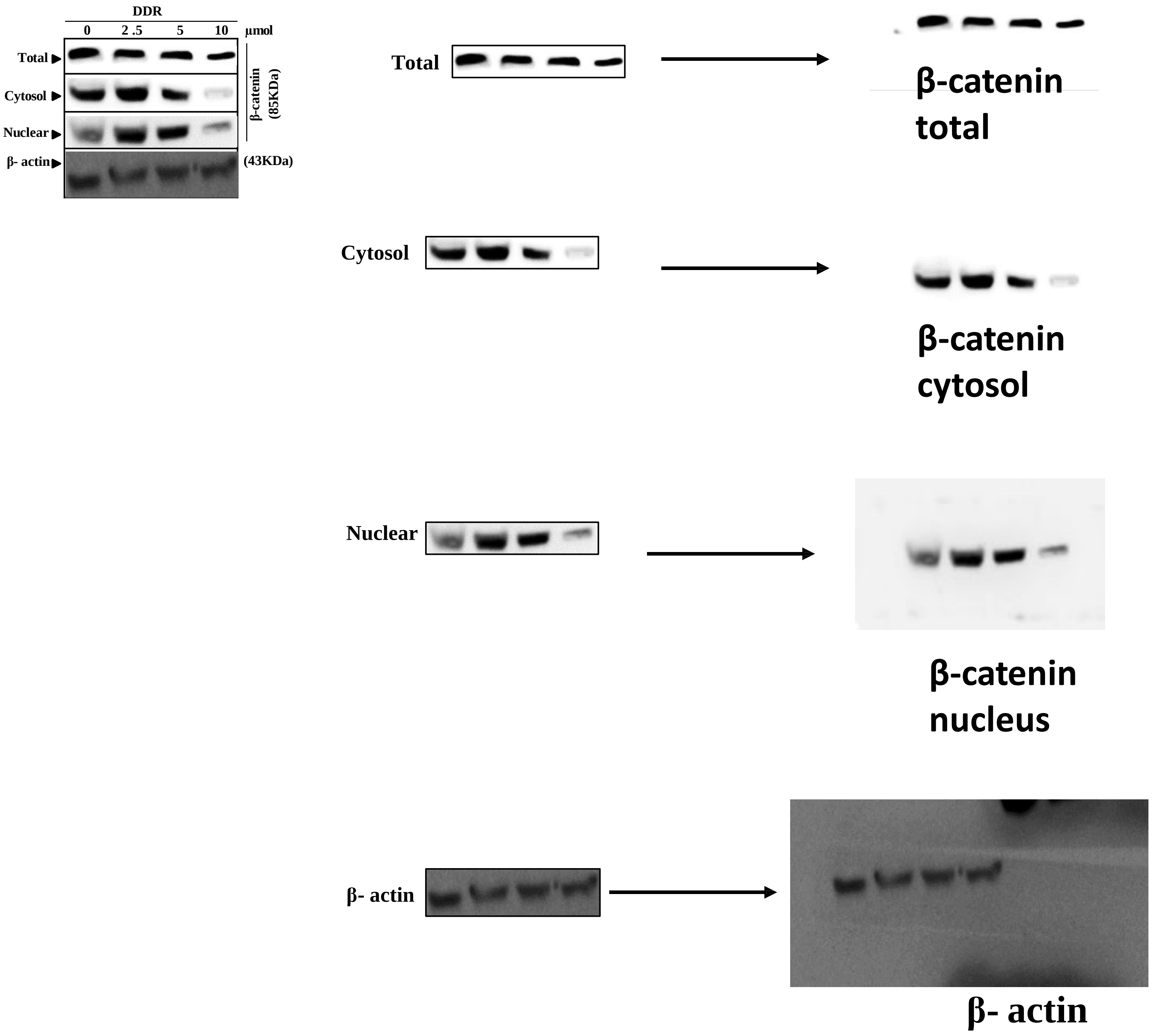
DDR
0
2 .5
5
10
µmol
Total
Cytosol
Nuclear
β- actin
β-catenin
(85KDa)
(43KDa)
Total
Cytosol
Nuclear
β- actin
β-catenin
total
β-catenin
cytosol
β-catenin
nucleus
β- actin

Figure 6 B

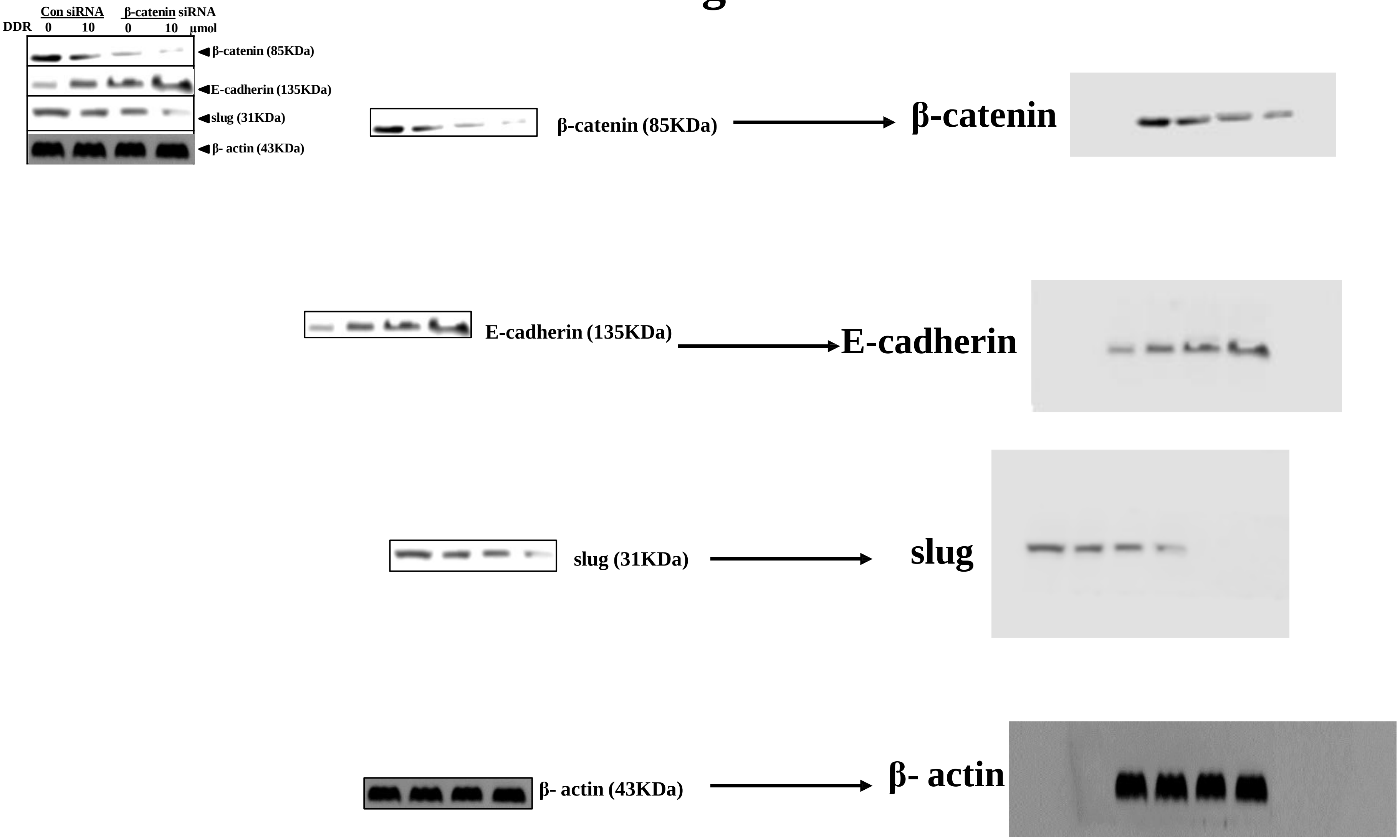

Con siRNA
β-catenin siRNA
DDR
0
10
0
10
μmol
β-catenin (85KDa)
E-cadherin (135KDa)
slug (31KDa)
β- actin (43KDa)
β-catenin (85KDa)
β-catenin
E-cadherin (135KDa)
E-cadherin
slug (31KDa)
slug
β- actin (43KDa)
β- actin

Figure 7

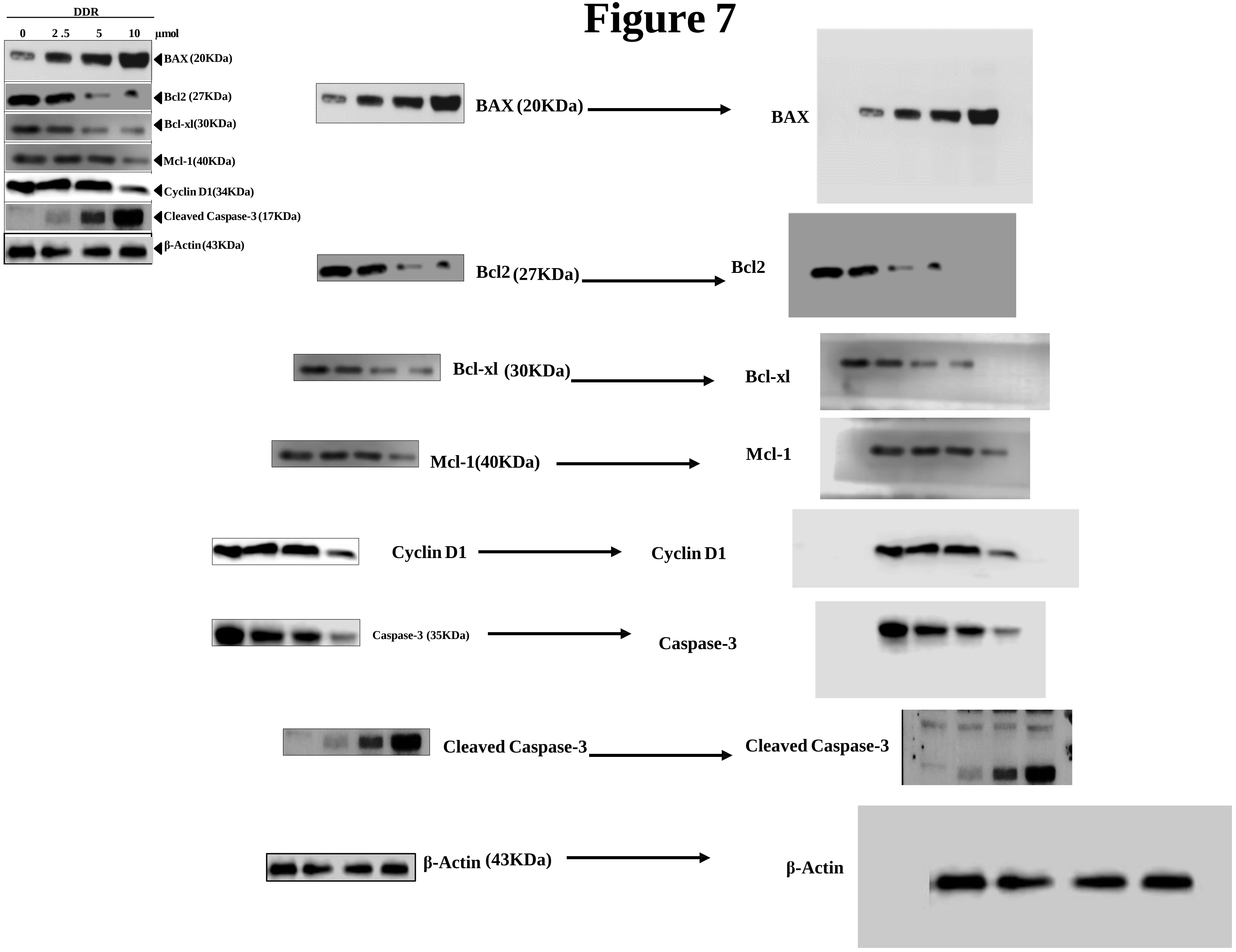
DDR
0
2 .5
5
10
µmol
BAX (20KDa)
Bcl2 (27KDa)
Bcl-xl(30KDa)
Mcl-1(40KDa)
Cyclin D1(34KDa)
Cleaved Caspase-3 (17KDa)
β-Actin (43KDa)
BAX (20KDa)
BAX
Bcl2 (27KDa)
Bcl2
Bcl-xl (30KDa)
Bcl-xl
Mcl-1(40KDa)
Mcl-1
Cyclin D1
Cyclin D1
Caspase-3 (35KDa)
Caspase-3
Cleaved Caspase-3
Cleaved Caspase-3
β-Actin (43KDa)
β-Actin
